# Metal-cation regulation of enzyme dynamics is a key factor influencing the activity of *S*-adenosyl-l-homocysteine hydrolase from *Pseudomonas aeruginosa*

**DOI:** 10.1038/s41598-018-29535-y

**Published:** 2018-07-27

**Authors:** Justyna Czyrko, Joanna Sliwiak, Barbara Imiolczyk, Zofia Gdaniec, Mariusz Jaskolski, Krzysztof Brzezinski

**Affiliations:** 1 Laboratory of Biochemistry and Structural Biology, Institute of Chemistry, University of Bialystok, Bialystok, Poland; 2 Center for Biocrystallographic Research, Institute of Bioorganic Chemistry, Polish Academy of Sciences, Poznan, Poland; 3 Laboratory of Biomolecular NMR, Institute of Bioorganic Chemistry, Polish Academy of Sciences, Poznan, Poland; 4 Department of Crystallography, Faculty of Chemistry, A. Mickiewicz University, Poznan, Poland

## Abstract

*S*-adenosyl-l-homocysteine hydrolase from *Pseudomonas aeruginosa* (PaSAHase) coordinates one K^+^ ion and one Zn^2+^ ion in the substrate binding area. The cations affect the enzymatic activity and substrate binding but the molecular mechanisms of their action are unknown. Enzymatic and isothermal titration calorimetry studies demonstrated that the K^+^ ions stimulate the highest activity and strongest ligand binding in comparison to other alkali cations, while the Zn^2+^ ions inhibit the enzyme activity. PaSAHase was crystallized in the presence of adenine nucleosides and K^+^ or Rb^+^ ions. The crystal structures show that the alkali ion is coordinated in close proximity of the purine ring and a ^23^Na NMR study showed that the monovalent cation coordination site is formed upon ligand binding. The cation, bound in the area of a molecular hinge, orders and accurately positions the amide group of Q65 residue to allow its interaction with the ligand. Moreover, binding of potassium is required to enable unique dynamic properties of the enzyme that ensure its maximum catalytic activity. The Zn^2+^ ion is bound in the area of a molecular gate that regulates access to the active site. Zn^2+^ coordination switches the gate to a shut state and arrests the enzyme in its closed, inactive conformation.

## Introduction

*S*-adenosyl-l-methionine (SAM) is a ubiquitous methyl group donor in methylation reactions of a wide range of acceptors including small and macromolecules^1^. The SAM-dependent reactions generate equimolar amounts of *S*-adenosyl-l-homocysteine (SAH), which is a strong inhibitor of SAM-dependent methyltransferases and needs to be removed to keep the metabolic processes going. By hydrolyzing SAH to adenosine (Ado) and L-homocysteine (Hcy), the enzyme SAHase (SAH hydrolase) serves as an important regulator of SAM-dependent methylation through the control of the SAM:SAH ratio, and is an indicator of the biological methylation activity of the cell^2–4^. SAHase inhibition or dysfunction leads to the accumulation of SAH in the cell and arrest of all crucial SAM-dependent processes. Apart from SAH hydrolysis, which is catalyzed in both directions, the enzyme also splits 2′-deoxyadenosine, which is an irreversible inhibitor of SAHases^5^. SAHase inactivation by 2′-deoxyadenosine proceeds *via* the formation of an unstable 3′-keto-2′-deoxyadenosine in an NAD^+^-dependent process that is very similar to the first step of the main SAH breakdown reaction. The subsequent and spontaneous cleavage of the N-glycosidic bond leads to the elimination of a modified sugar moiety, while the adenine molecule stays bound in the active site of the enzyme^6^. The reduced cofactor (NADH) is not regenerated in the 2-deoxyadenosine reaction, stalling the enzyme in the inactive form.

SAHases are usually active as homotetramers^7–15^. The protomer has a three-domain fold, with two prominent domains, the substrate- and cofactor-binding domains, separated by a deep crevice. A small C-terminal dimerization domain locks two subunits into a homodimer by quaternary complementation of the cofactor binding site, where a tightly but non-covalently bound nicotinamide adenine dinucleotide (NAD^+^) is found in its oxidized form. During the catalytic SAHase cycle, the cofactor is first reduced upon abstraction of a hydride anion from the C3′ atom of the sugar moiety. To complete the cycle, the NAD^+^ cofactor is regenerated through Michael-type addition of a water molecule^16^. The latter step is not part of the irreversible inactivation of the enzyme by 2′-deoxyadenosine, in which case the cofactor is trapped in its non-reactive, reduced state^5,6^. The enzyme undergoes a significant open-closed conformational transformation upon substrate or inhibitor binding. A comparison of the enzyme conformation in the open and in the closed forms reveals that the interdomain crevice is constricted upon substrate or inhibitor binding. The accessibility of the active site from the solvent region is regulated by a molecular gate formed by a conserved pair of His-Phe residues^6,10,11^. Depending on the conformational state of the gate, the channel leading to the active site can be open or shut. The structures of SAHases from various species were determined mainly for the enzymes in the closed conformation, in the presence of adenosine (Ado) or its analogs. In most of those structures, various monovalent cations were found in a coordinating loop located in close proximity of the substrate binding site. However, the ions do not interact with the ligand directly. The type of the coordinated ion strongly depends on the crystallization conditions, which included NH_4_^+^, Na^+^ or K^+^ cations. Only a few SAHase structures correspond to the open form^8,11^, which is correlated with the absence of an active-site ligand, as well as of a monovalent cation in the substrate binding region. This suggests that the cation is coordinated upon a conformational transformation of the enzyme during the catalytic cycle. However, it is still unclear which monovalent cation is utilized by the enzyme under physiological conditions and what role it serves in the catalytic process. Additionally, it has been suggested that SAHase activity could be regulated by some divalent cations, e.g., copper^17^. However, so far none of the previously determined SAHase structures contained any metal cations other the monovalent ones^6,7,9–11,14,15,18,19^.

*Pseudomonas aeruginosa* has natural resistance to many antibiotics and disinfectants and is responsible for numerous infections. Therefore, defining new molecular targets for the treatment of *P*. *aeruginosa* infections is of high medical priority. The purpose of the present study has been to define the catalytic role of the monovalent cations and to explain the molecular mechanism of *P*. *aeruginosa* SAHase (PaSAHase) inhibition by zinc ions. By combining X-ray crystallography with enzyme kinetics and ITC studies, as well as with ^23^Na NMR spectroscopy, we were able to elucidate the effect of the monovalent cations on ligand binding, and to explain that the enzyme is most efficient in the presence of potassium. Also, we confirmed the presence of zinc ions by X-ray fluorescence and explained how Zn^2+^ binding blocks a molecular gate, leading to a complete inhibition of enzymatic activity.

## Results

### Overall structure of PaSAHase

We present four crystal structures of PaSAHase/ligand/ion(s) complexes at resolutions of 1.35 to 1.75 Å (Supplementary Information Table S1). Two complexes of Zn^2+^-containing PaSAHase were crystallized in the presence of K^+^ ions and adenosine (Ado/K^+^/Zn^2+^), or an inhibitor, 2′-deoxyadenosine (2′-dAdo/K^+^Zn^2+^). Additionally, the latter ligand as well as 3′-deoxyadenosine (non-metabolized adenosine analog) were used for crystallization of zinc-free enzyme in the presence of Rb^+^ (2′-dAdo/Rb^+^) or K^+^ (3′-dAdo/K^+^) cations. In the Zn^2+^-containing complexes, the divalent cation site is not fully occupied. Despite the very low activity of the enzyme in the presence of Zn^2+^ cations, 2′-deoxyadenosine undergoes catalytic cleavage at the N-glycosidic bond during incubation with the enzyme or during crystallization^6^. Therefore, only adenine is found in the active site of PaSAHase crystallized in the presence of 2′-deoxyadenosine and K^+^ or Rb^+^ cations. It is of note, that the absence of sugar moiety does not change the spatial arrangement of the active site when compared to the complexes with nucleosides. This results from the presence of a phosphate anion from a crystallization buffer, which mimics the positions of the oxygen atoms of the ribose moiety. A similar effect was noted previously^6,19^, when four water molecules, superposable with the oxygen atoms of the sugar moiety, were observed close to the adenine molecule. The four water molecules, as well as the phosphate anion are involved in a hydrogen bond network that is very similar to that observed in nucleoside complexes. The presence of water molecules or a phosphate ion depends only on the crystallization conditions. Thus, the anion is not required for adenine binding and to stabilize the closed conformation of the protein. Moreover, the molecule that binds directly to the enzyme is 2′-deoxyadenosine, which is then slowly decomposed to adenine and a sugar moiety^5^. It should be noted that SAHase enzymatic activity assays are carried out in phosphate buffer, as this anion has no inhibitory effect on SAHase activity^20–22^.

**Figure 1:**
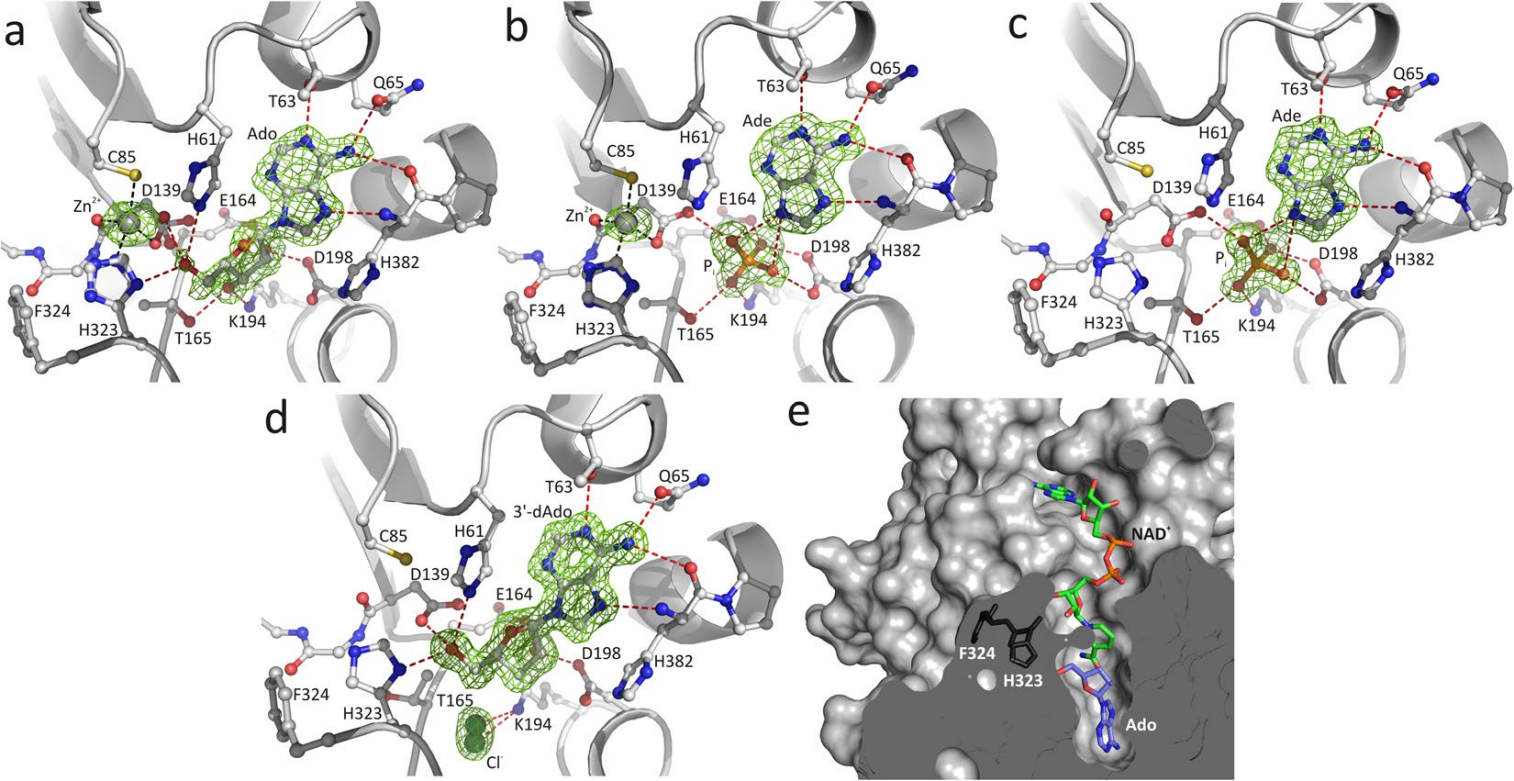
The mode of ligand binding in the substrate binding site of PaSAHase crystallized in the presence of: (**a**) adenosine, K^+^ and Zn^2+^ ions; (**b**) 2′-deoxyadenosine, K^+^ and Zn^2+^ ions; (**c**) 2′-deoxyadenosine and Rb^+^ ions; (**d**) 3′-deoxyadenosine and K^+^ ions. Possible hydrogen (red) and coordination (black) bonds are indicated by dash lines. Zn^2+^ and Cl^-^ ions are represented by gray and green spheres, respectively. The *mF*_*o*_*-DF*_*c*_ difference electron density OMIT maps are contoured at 3.0σ. (**e**) The accessibility of the active site from the solvent region depends on the state of the molecular gate formed by H323-F324. In all four complexes (here illustrated by subunit A of the Ado/K^+^/Zn^2+^ complex) the channel is shut and the ligand molecule is trapped in the substrate-binding site.

The binding modes of adenine and its nucleosides in the active site of PaSAHase are similar to those observed in other SAHase models of various origin^6,7,9–11,14,15,18,19,23,24^, as shown in Fig. 1a–d. The polar interactions in the active site of the enzyme in all four complexes are presented in Supplementary Information (Table S2**)**. It is of note that in the presented PaSAHase complexes, adenine or its nucleosides are trapped in the active site, as the H323-F324 molecular gate is shut **(**Fig. 1e**)**.

**Figure 2:**
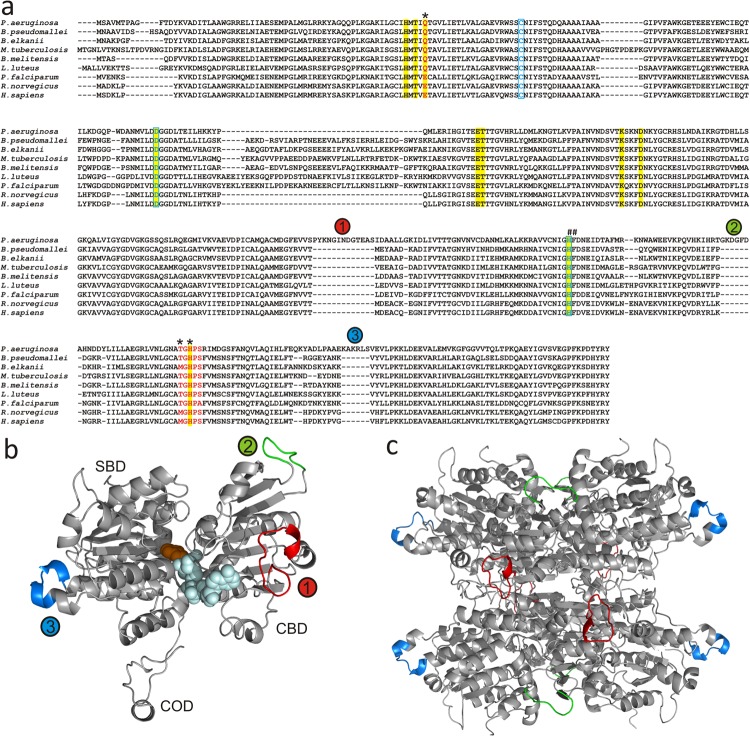
(**a**) Multiple sequence alignment of selected SAHases. Amino acid residues marked in red are in the monovalent ion binding region and the asterisks indicate residues directly involved in metal coordination. A tandem of His-Phe residues (marked with #) form a molecular gate that regulates the accessibility of the active site from the solvent region. Residues involved in substrate binding are highlighted in yellow. Residues involved in the coordination of Zn^2+^ ions are marked in blue and boxed. Circled numbers relate to lineage-specific insertions located in the cofactor- (1,2) and substrate-binding (3) domains of PaSAHase. (**b**,**c**) Overall structure of PaSAHase. (**b**) The structure of PaSAHase subunit with color-coded specific insertions located in the cofactor-binding domain (CBD), residues 270–280 (1) and 353–359 (2) shown in red and green, respectively, and in the substrate binding domain (SBD), residues 414–419 (3) shown in blue; COD is small C-terminal oligomerization domain. The NAD^+^ cofactor (pale blue) and adenosine (orange) are in space-filling representation. (**c**) A ribbon diagram of the active tetrameric form of PaSAHase, with the insertions marked by color as in (**b**).

PaSAHase forms a homotetramer in solution. The complexes crystallize in two different forms, either as a tetramer or as a dimer from which the complete homotetramer is generated by crystallographic symmetry. All the tetramers are very similar, with r.m.s. deviations of their Cα superpositions ranging from 0.08–0.35 Å. A multiple sequence alignment of amino acid sequences of different SAHases (Fig. 2a) reveals a lineage-specific insertion segments in PaSAHase, located in the substrate (inserted residues 414–419) and cofactor (inserted residues 270–280 and 353–359) binding domains on the surface of the enzyme (Fig. 2b,c).

### The monovalent cation near the active site of PaSAHase

**Figure 3:**
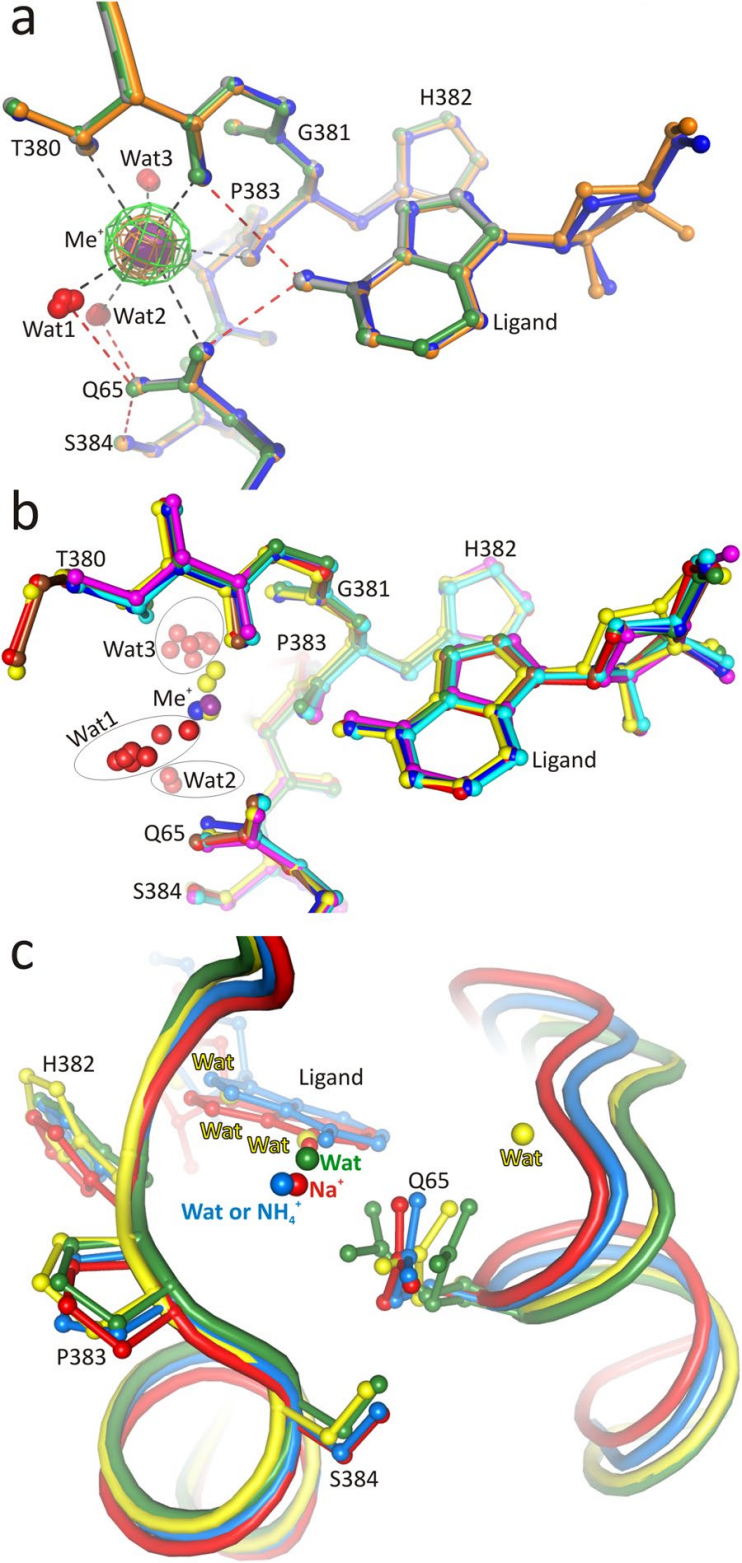
Superpositions of the monovalent cation coordination site and the ligand bound in the substrate binding site of SAHase models. (**a**) Superposition of PaSAHase complex models, Ado/K^+^/Zn^2+^ (orange), 2′-dAdo/K^+^/Zn^2+^ (gray), 3′-dAdo/K^+^ (blue), and 2′-dAdo/Rb^+^ (green). The red and purple spheres indicate water molecules and monovalent cations, respectively. The difference *mF*_*o*_*-DF*_*c*_ OMIT map (green) for the potassium cation (Ado/K^+^/Zn^2+^) and anomalous difference map for the Rb^+^ cation (orange) are contoured at 3.0σ and 25.0σ, respectively. Possible coordination (black) and hydrogen (red) bonds are indicated by dash lines. (**b**) Superposition of the monovalent cation coordination region of PaSAHase (cyan) with models of the enzymes from *Homo sapiens* (yellow, PDB entry 1LI4), *Plasmodium falciparum* (blue, 1V8B), *Brucella melitensis* (magenta, 3N58), *Lupinus luteus* (green, 3OND), *Bradyrhizobium elkanii* complexed with Na^+^ (red, 5M5K), and NH_4_^+^ (brown, 4LVC). The Cα superpositions are based on chains A of the models. Potassium (purple), sodium (yellow) and ammonium (blue) cations, as well as water molecules (red) are represented as spheres. Three water clusters, which correspond to three (Wat1-Wat3) water molecules involved in potassium coordination, are encircled. (**c**) Superposition of the monovalent cation coordination region of the enzyme in the closed conformation (red, *B*. *elkanii*, PDB entry 5M5K, chain A) with models of the enzyme in semi-open conformation (blue, *B*. *elkanii*, 5M5K, chain D) and open conformation (green, *B*. *elkanii*, 4LVC, chain D; and yellow, *B*. *melitensis*, 3N58, chain B). Sodium and ammonium ions, as well as water molecules are represented by spheres. Residue numbers correspond to PaSAHase sequence.

In all presented PaSAHase complexes, the four subunits exist in the closed conformation and each of them binds an adenine nucleoside or adenine, as well as an alkali metal cation, K^+^ or Rb^+^, in the substrate binding area (Fig. 3a). The K^+^/Rb^+^ cations are bound in the monovalent cation coordinating loop (T380-S384) in close proximity of the purine ring of the ligand, but they do not interact with the ligand directly. Additionally, using anomalous dispersion, one non-specific Rb^+^ site per PaSAHase tetramer was found as a crystallization artifact in the rubidium complex. The metal coordination loop is part of one of the two hinge regions linking the substrate- and cofactor-domains that are involved in substrate-induced domain movements^8,23,24^. The coordination sphere of the K^+^/Rb^+^ cations is a slightly distorted pentagonal bipyramid (Supplementary Information Table S3) formed by two main-chain carbonyl oxygen atoms (T380 and H382) and the side-chain oxygen atoms of T380 and a highly conserved Q65 residue involved in ligand binding. Three water molecules complete the coordination sphere. A very similar coordination geometry of the K^+^ cation was observed in the crystal structure of SAHase from *Brucella melitensis* (PDB ID: 3N58, unpublished). Other SAHase models deposited in the PDB bind NH_4_^+^ or Na^+^ cations at this position. Moreover, in numerous PDB models this position is marked as a water molecule, while in fact it is an alkali metal cation, most likely Na^+^, coordinated by at least five oxygen atoms. Despite the presence of different monovalent cations, the conformation of the coordinating loop and of the Q65 residue is not affected to any significant degree; likewise, the position of the ligand molecule is very similar in all analyzed models (Fig. 3b). The presence of cations other than potassium is usually reflected in a different arrangement of the coordination sphere (Supplementary Information Table S4). Most importantly, the amide oxygen of the highly conserved Q65 residue interacts with NH_4_^+^ or Na^+^ ions indirectly, *via* a water molecule^6,10,11^, with one notable exception (PDB ID: 5M5K) where the glutamine side chain is involved in direct sodium coordination^19^.

### Effect of monovalent cations on PaSAHase activity

**Figure 4:**
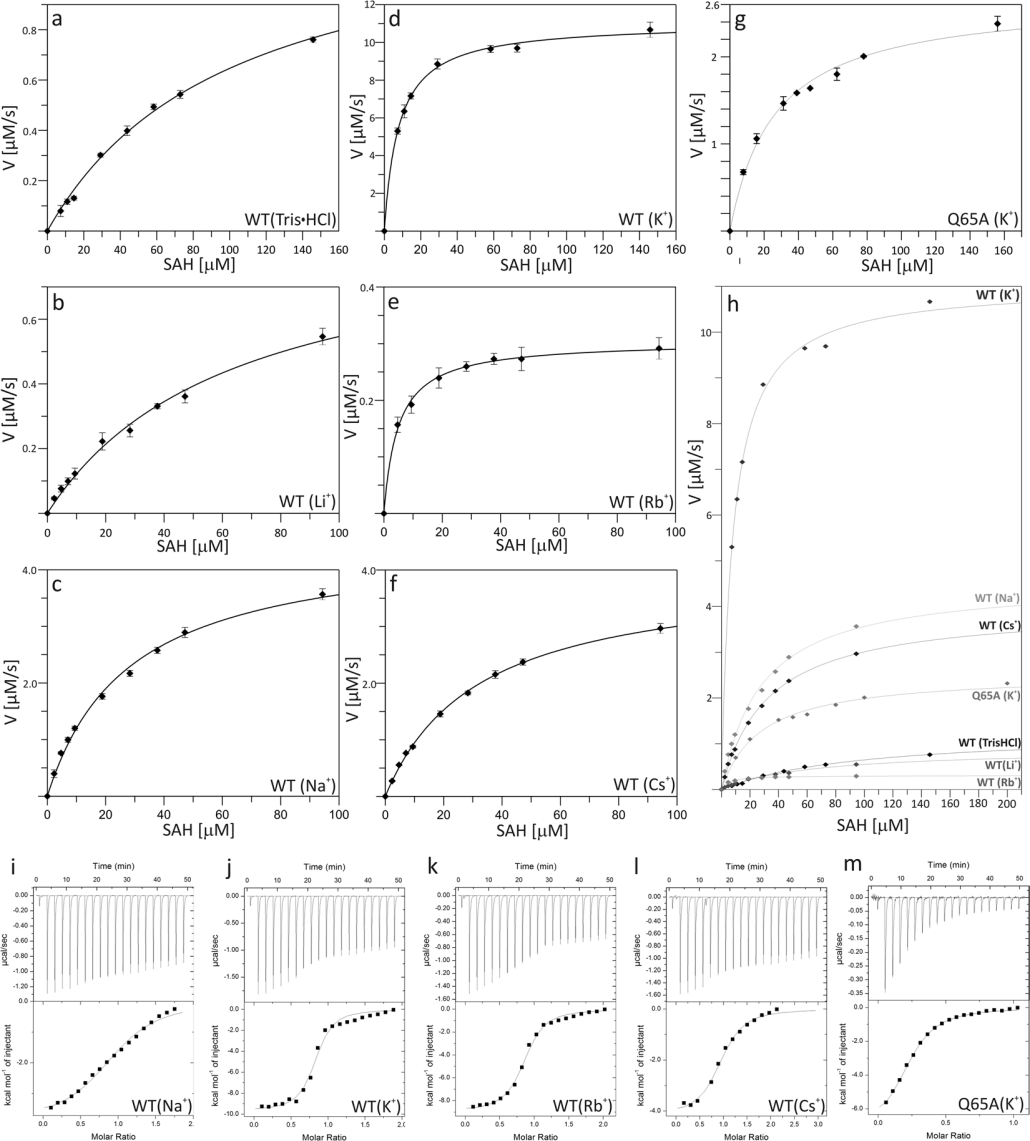
Influence of monovalent cation type and Q65A mutation on enzyme activity and ligand binding. Kinetic analysis of wild-type PaSAHase (**a**–**f**) and its Q65A mutant (**g**) were conducted in the presence of 100 mM Tris·HCl (**a**), Li^+^ (**b**), Na^+^ (**c**), K^+^ (d and g), Rb^+^ (**e**) or Cs^+^ (**f**) cations. The activity was determined using variable concentrations of S-adenosyl-l-homocysteine (SAH). In panel (h) all the kinetic curves are presented on common scale to emphasize the differences in enzymatic activities. Calorimetric titrations of PaSAHase with adenosine were conducted in the presence of 100 mM alkali cations: Na^+^ (**i**), K^+^ (**j**), Rb^+^ (**k**), and Cs^+^ (**l**) and its Q65A mutant in the presence of K^+^ ions (m). The top plot of each panel represents the raw heat data obtained from consecutive injections of adenosine into the sample cell containing the protein. The heat peak areas have been plotted against the molar ratio of adenosine added to the enzyme, creating the binding isotherm at the bottom of each panel. The best fit of the model of one set of binding sites is represented by the solid line. All titrations were performed at 293 K.

The crystal structures of SAHases available so far contain NH_4_^+^, Na^+^ or K^+^ ions near the substrate binding site, depending on the composition of the purification and crystallization media. The obvious candidate for the monovalent cation involved in the enzymatic activity of SAHase under physiological conditions is potassium, which is the major cation of all living cells and is also a common metal cation bound to cellular proteins^25^. Within this project, enzyme kinetics and isothermal titration calorimetry (ITC) studies were conducted in the presence or absence of alkali cations to investigate their influence on the enzymatic activity and ligand binding of PaSAHase. (Tables 1, 2 and Fig. 4). In the kinetic experiments, the highest activity was observed in the presence of K^+^ cations. The catalytic rate (k_cat_) of the reaction in the presence of K^+^ is 0.575 ± 0.009 s^−1^ and is 2–11 times higher when compared to the rates measured in the presence of other alkali ions or without them. In the case of Rb^+^, the drop of k_cat_ is almost 33-fold. Moreover, for most of the reactions, the substrate affinity (K_M_) is 3–12 times higher in the presence of potassium. The notable exception is the reaction in the presence of Rb^+^, where the measured substrate affinity is slightly higher than for potassium. Finally, the catalytic proficiency index k_cat_/K_m_ is one to over two orders of magnitude higher in the presence of potassium than for other ions. These findings clearly demonstrate that the enzyme attains its maximum catalytic activity when the K^+^ cation is bound. On the other hand, Rb^+^ acts as noncompetitive inhibitor.

**Table 1:** Kinetic parameters characterizing the enzymatic activity of wild type (WT) and Q65A PaSAHase in the absence or presence of alkali metal cations.

Enzyme/Alkali cation	V_max_ [µM·s^−1^]	k_cat_ [s^−1^]	K_M_ [µM]	k_cat_/K_M_ [M^−1^·s^−1^]
WT/none	(16.9 ± 0.9).10^−3^	(52.9 ± 3.0).10^−3^	101.1 ± 9.9	523 ± 81
WT/Li^+^	(14.9 ± 1.1).10^−3^	(46.6 ± 3.3).10^−3^	63.8 ± 8.0	731 ± 143
WT/Na^+^	(76.0 ± 2.6).10^−3^	(237.3 ± 8.0).10^−3^	28.0 ± 2.2	8467 ± 940
WT/K^+^	(184.0 ± 2.1).10^−3^	(575.0 ± 9.0).10^−3^	7.9 ± 0.4	72502 ± 4858
WT/Rb^+^	(5.6 ± 0.1).10^−3^	(17.5 ± 0.3).10^−3^	4.9 ± 0.3	3568 ± 237
WT/Cs^+^	(68.2 ± 1.1).10^−3^	(213.1 ± 3.4).10^−3^	32.0 ± 1.1	6668 ± 333
Q65A/K^+^	(44.7 ± 1.5).10^−3^	(140.0 ± 4.7).10^−3^	26.2 ± 2.6	5322 ± 358

The values were obtained as averages of two replicates, ± represents standard error of the mean (SEM) values.

**Table 2:** Thermodynamic parameters obtained from ITC titrations of wild type (WT) and Q65A PaSAHase with adenosine in the presence of monovalent cations.

Parameter	WT/Na^+^	WT/K^+^	WT/Rb^+^	WT/Cs^+^	Q65A/K^+^
N	0.97 ± 0.02	0.80 ± 0.01	0.84 ± 0.01	0.94 ± 0.02	0.236 ± 0.004
K_d_ [µM]	8.8 ± 1.0	1.2 ± 0.2	1.8 ± 0.2	4.7 ± 0.6	7.3 ± 0.4
ΔH [cal/mol]	−3891 ± 114	−9665 ± 237	−8935 ± 137	−4174 ± 115	−7447 ± 167
ΔS [cal/mol/°]	9.8	−5.9	−4.2	10.1	2.0

The parameters obtained from sigmoidal curve fitting are given with ± standard deviations as follows: stoichiometry (N), dissociation constant (K_d_), changes in enthalpy (ΔH) and entropy (ΔS).

Also the ITC experiments indicate the importance of K^+^ cations for ligand (adenosine) binding. During the titrations conducted in the absence of any alkali metals or the presence of Li^+^ ions, the heat effect was immeasurably small. For the experiments conducted in the presence of Na^+^, K^+^, Rb^+^ and Cs^+^ ions, the stoichiometry of adenosine binding to the enzyme is one ligand molecule per subunit. The optimal cation for PaSAHase-Ado binding is K^+^, as the ligand binding is mostly enthalpy driven and the binding constant K_d_ of 1.2 ± 0.2 µM is the lowest in the presence of potassium. Adenosine binding is only slightly weaker in the presence of Rb^+^. However, the use of Na^+^ or Cs^+^ cations resulted in a significant decrease of binding specificity and increase of entropy contribution (Table 2).

### Formation of the monovalent cation binding site

The majority of the crystallographic models of SAHases in the PDB present the enzyme in the closed conformation with a ligand and a monovalent cation bound in the substrate binding region. A few structures present a situation where one or all four subunits of the tetrameric enzyme do not bind a ligand and, therefore, adopt the open conformation^8,11^. In one case, a subunit does contain a ligand (3′-deoxyadenosine) but is found in a semi-open conformation, intermediate between the open and closed states^19^. Those models were derived from crystals that were grown in the presence of NH_4_^+^, Na^+^ or K^+^ cations. Inspection of the subunits that are in the open conformation reveals that there is no monovalent cation bound near the substrate binding site, although the metal coordination loop has the same conformation as in the subunits with the closed conformation (Fig. 3c). Also, a very similar loop conformation is observed for the subunit in the semi-open state, however, an undifferentiated water molecule/ammonium ion is located in the position of the monovalent cation. The major structural difference between the closed/semi-open and open states concerns the conformation of Q65 (PaSAHase numbering). In the open state this residue is located far away from the metal binding loop and its side chain is disordered in at least two conformations. Thus, the crystallographic models suggest that the coordination of the monovalent cation does not occur until a conformational transformation upon ligand binding in the active site has taken place. This postulate was confirmed by two other experiments. (i) No effect other than K^+^ dilution heat was observed during the isothermal titration of a ligand- and alkali-metal-free PaSAHase with K^+^ ions. This result suggests that the cation does not bind specifically to the enzyme in its open (i.e., ligand-free) conformation. (ii) In ^23^Na NMR spectra recorded for either adenosine or PaSAHase in the presence of 5 mM NaCl, only one peak is observed with a chemical shift as for NaCl itself (Supplementary Information Fig. S1a). This shows that, in general, the environment of the sodium nuclei is the same in all three samples. The NMR results are in agreement with the isothermal titration data and confirm that monovalent cations (at least Na^+^) do not bind specifically to the open form of the protein. The observed broadening of the ^23^Na resonance in the sample containing PaSAHase indicates that non-specific Na^+^-protein interactions cannot be excluded. The situation is changed when the spectra are recorded for samples containing both the enzyme and the adenosine ligand in the presence of Na^+^ ions. In this scenario, PaSAHase should adopt the closed conformation enforced by adenosine binding, allowing the cation to be coordinated near the ligand, as indicated by the crystallographic results. In agreement with this scenario, a small but meaningful shift is observed in the ^23^Na NMR spectrum of PaSAHase/adenosine/^23^Na^+^. This effect increases with a decrease of the Na^+^:PaSAHase ratio (Supplementary Information Fig. S1b) owing to the increased proportion of protein-coordinated Na^+^ ions in the sample and a concomitant contribution of the bound form to the position of the ^23^Na signal, which is the weighted average of two signals in fast exchange, derived from the nuclei of protein-coordinated and unbound/hydrated Na^+^ ions.

In view of all the above observations, the emerging course of events is that the K^+^ ion coordination site is formed only upon ligand binding, when the enzyme changes its conformation from the open to the closed state. This site is composed of the highly conserved Q65 residue as well as of the loop region T380-S384 that links the substrate- and cofactor-binding domains, with T380 and H382 involved in direct cation coordination. Interestingly, the conformation of the loop does not change significantly during this transformation (Fig. 3c). The situation at the Q65 residue is drastically different. In the open form of the enzyme, the Q65 residue is far from the loop region and its side chain is disordered. Upon ligand binding, Q65 moves close to the coordination loop as the result of the movement of the substrate-binding domain towards the cofactor-binding domain, so that its side chain can directly coordinate the potassium cation. In addition, metal coordination stabilizes and orders the side chain of Q65. With the Oε atom coordinated to the metal cation, the amide group of Q65 is now adequately oriented for the formation of an Oε…N hydrogen bond with the exoamino group of the adenine moiety of the ligand. The importance of Q65 in ligand binding and enzymatic activity of PaSAHase is reflected in our mutagenesis study. In the presence of K^+^ ions, the Q65A mutant is over four times less active and the K_M_ value increases over three times compared to the wild type enzyme. The catalytic proficiency index k_cat_/K_m_ is over one order of magnitude lower. ITC titrations also confirmed the importance of the Q65 residue for the catalytic cycle. During the titrations of the Q65A mutant with adenosine in the presence of K^+^, the stoichiometry of adenosine binding to the enzyme is ~0.25, indicating that, on average, three substrate binding sites of the tetramer remain empty. Finally, more than six-fold drop of adenosine affinity is observed in comparison to native PaSAHase. The kinetic and thermodynamic measurements are presented in Fig. 4 and in Tables 1 and 2.

### The highly conserved glutamine residue and its rare substitutions

Phylogenetic analyses of SAHase sequences indicate that the enzymes diverged into two major groups during their evolution^26^. The first group comprises enzymes from bacterial and eukaryotic organisms while the second is formed by archaeal SAHases. The consequences of this divergent evolution are also visible at the structural level. In particular, the mode of adenine ring binding in the substrate domain is different in SAHases from different species. The Q65 residue is highly conserved among bacterial and eukaryotic sequences (Fig. 2a). Phylogenetic analyses suggest that the primordial residue that interacted with the adenine amine group was indeed glutamine. Among eukaryotic SAHases, only in mammalian (e.g., human) and some protozoan (e.g., the pathogenic strains of *Plasmodium*, *Cryptosporidium*, *Theileria* and *Naegleria*) enzymes this glutamine residue is substituted by glutamate. It is of note that these protozoan pathogens are obligate intracellular parasites of humans and other animals with the same substitution. This might suggest co-evolution (but not horizontal gene transfer, as these sequences belong to distant branches of the phylogenetic tree) of the host-pathogen pair with the further implication that the presence of glutamate instead of glutamine at this position is a relatively new evolutionary feature in the first group of SAHases. The crystal structures of several mammalian and protozoan SAHases that carry the Gln→Glu substitution were solved in the presence of Na^+^ or NH_4_^+^ cations^9,14,27^. With this substitution, the glutamate carboxylic oxygen can interact directly, or indirectly *via* a water molecule, with the Na^+^ cation, analogously to the amide oxygen of the glutamine side chain (Supplementary Information Table S4). However, the alkali metal cation is still required for the glutamate side chain ordering. Among SAHases from the second, archaeal group, this position can be occupied either by glutamine, glutamate, or sometimes by a lysine residue. In the latter case the lysine amino group would have to be unprotonated in order to act as an acceptor of the hydrogen bond from the exoamino group of adenine. Moreover, a monovalent cation cannot be involved in the positioning of the lysine side chain. Indeed, the structure of archaeal-type SAHase from *Thermotoga maritima* crystallized in the presence of the SAH substrate does not contain any metal cation in the substrate binding region^12^.

### Potassium cations ensure the highest activity through the regulation of enzyme dynamics

The crystallographic studies of SAHases show that the enzymes can exist in at least two distinct conformations, i.e., in the open (ligand-free) and closed (ligand-bound) state. On the other hand, measurements of fluorescence anisotropy (conducted for enzymes with a covalently attached fluorescence probe) in solution revealed that the domain oscillations occur also in substrate-free SAHase^24,28^. These results indicated that the enzyme exists in an equilibrium of the open and closed states, shifted in the direction of the of the substrate-free open form. The frequency of the oscillations was estimated^28^ at ~4 × 10^7^ s^−1^. This value exceeds by several orders of magnitude the diffusional-encounter frequency of the enzyme^27^, and by 4–8 orders of magnitude the catalytic rates (k_cat_) of SAHases from various sources^29^. Confrontation of these numbers could give the impression that the probability of binding of the substrate and its conversion to products, which ultimately need to be released, is very low. However, in structural studies, binding of the substrate induces the enzyme to change its conformational from the open to the closed state. It is essential that the enzyme should not be frozen in the closed state, but stayed in it only as long as is required for the catalytic reaction to complete, and then proceeded to the open conformation for product release and binding of another substrate molecule. At this point the exact role of the K^+^ ions in the enzymatic activity cannot be fully explained. The cation/ligand binding regions are not affected structurally by the type of the coordinated monovalent cation, but obviously there are significant cation-dependent differences in the thermodynamics of the ligand binding and enzyme kinetics.

**Figure 5:**
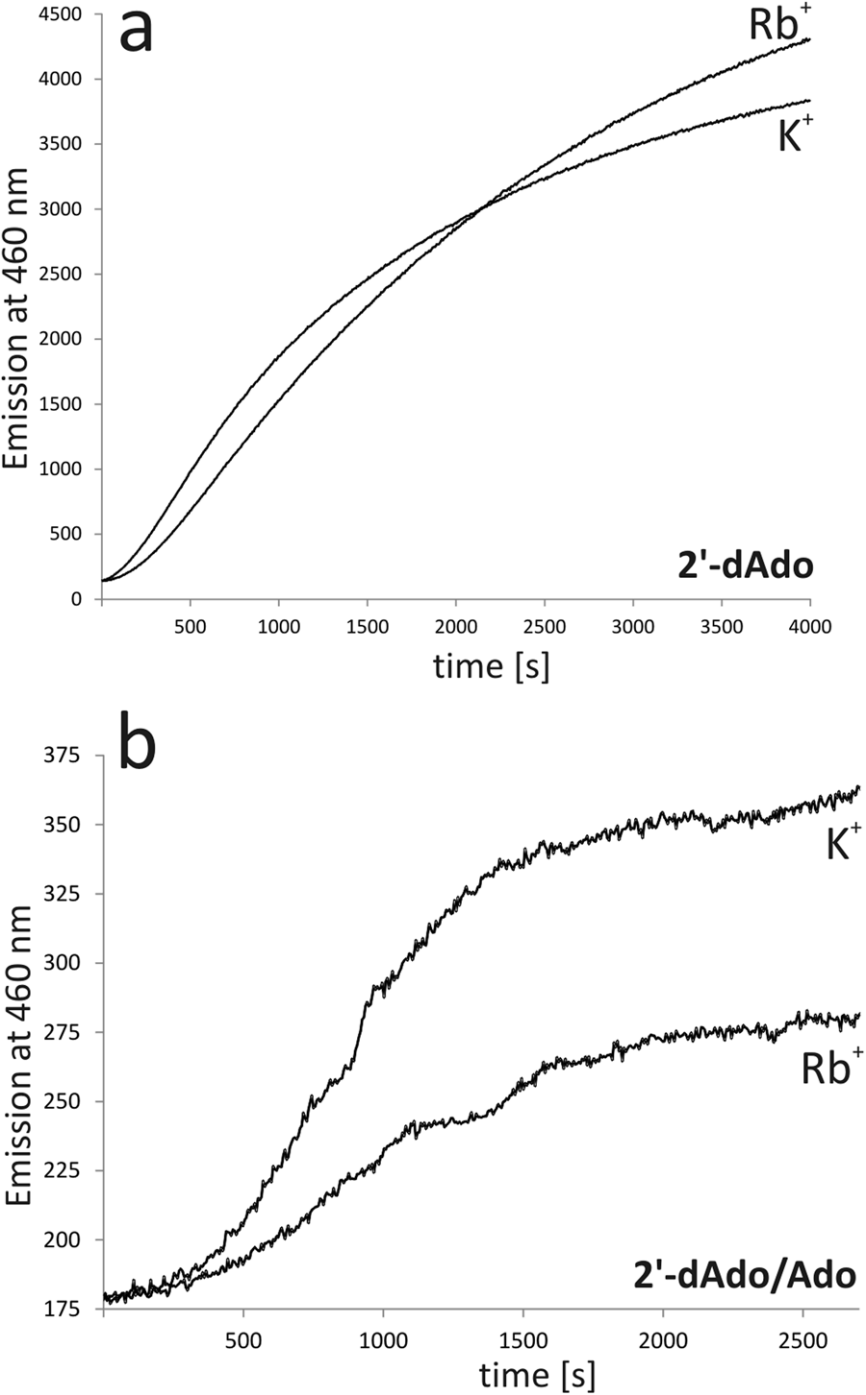
(**a**) Time course of PaSAHase inactivation by 2′-deoxyadenosine (2′-dAdo) in the presence of K^+^ or Rb^+^ ions. (**b**) Time course of inactivation of PaSAHase incubated with adenosine (Ado) by 2′-deoxyadenosine in the presence of K^+^ or Rb^+^ ions. Irreversible formation of the reduced form of the cofactor (NADH) was monitored spectrofluorimetrically after the addition of the competitive inhibitor 2′-deoxyadenosine.

To evaluate this puzzling effect of alkali cations on SAHase activity, two experiments were performed, using 2′-deoxyadenosine, an inhibitor that is involved in the irreversible reduction of the NAD^+^ cofactor to NADH during the chemical conversion of the inhibitor to adenine and a sugar residue^5,6^. In the first experiment, PaSAHase was incubated in the presence of 2′-deoxyadenosine in a buffer containing Rb^+^ or K^+^ ions. In the second experiment, the enzyme was first incubated with adenosine in the presence of Rb^+^ or K^+^ and subsequently 2′-deoxyadenosine was added. In both experiments, the rate of NADH formation was monitored spectrofluorimetrically (Fig. 5a,b). In the first experiment at its initial stage, the rate of NADH formation was only slightly higher for the reaction conducted in the presence of K^+^, however, the intensity of fluorescence emission reached a plateau faster in the presence Rb^+^ ions, indicating that in the time course the SAHase_closed_-Ligand-Rb^+^ complex forms more easily and/or the ligand is bound more tightly. In the second experiment, the rate of NADH formation was significantly lower in the presence of Rb^+^. Moreover, in the presence of Rb^+^ the fluorescence emission plateau was reached at lower intensity, indicating lower content of the reduced form of the cofactor. This result clearly indicates that the SAHase_closed_-Ligand-Rb^+^ complex is more stable than SAHase_closed_-Ligand-K^+^, as the exchange of the bound adenosine to 2′-deoxyadenosine is hampered in the complex containing the Rb^+^ ions.

The above experiments strongly suggest that for the explanation of the role of potassium in the catalytic activity of SAHase, one should consider the stability of the enzyme-ligand-cation complex (SAHase_closed_-Ligand-Me^+^). Assuming the same ligand (e.g., adenosine) is bound in the active site, the stability of such a complex would depend only on the metal cation. Coordination of the alkali ion by protein and water oxygen atoms can be treated as a hydration-like process, involving ion-dipole interactions. From the thermodynamic point of view, a decrease in the absolute value of the enthalpy of ion hydration is correlated with the increase of the ionic radius and indicates stronger ion-dipole interactions with smaller ions. However, this is not the case in the SAHase_closed_-Ligand-Me^+^ complex, where the coordination region is rigid with very similar, cation-independent conformation found in different SAHase models (Fig. 3b). The cation is involved in the formation of a network of polar contacts including the coordinating loop (residues T380-S384, part of the molecular hinge), the side chain of Q65, the substrate/ligand, and several water molecules (Fig. 3a). The ion coordination region is involved in ligand binding and seems to be ideally suited to coordinate K^+^ and Rb^+^, the two ions tested that have similar ionic radii (1.46 vs 1.56 Å, coordination number VII)^30^. This conclusion is confirmed by the ITC study, which revealed that binding of the adenosine ligand is very similar and most efficient in the presence of these two cations. On the other hand, their influence on enzyme activity is completely opposite. The ionic radius of Rb^+^ is slightly larger than K^+^ and, therefore, it should form a more stable SAHase_closed_-Ligand-Me^+^ complex, as the contribution of the coordination bond to the protein/water O–Rb^+^ interaction is higher than for the complex with K^+^. In consequence, dissociation of the SAHase_closed_-Ligand-Rb^+^ complex is more difficult and the enzyme is arrested in its closed state. In this scenario, the Rb^+^ ions inhibit the enzyme activity in a noncompetetive manner. In the SAHase_closed_-Ligand-K^+^ complex, the contribution of the coordination bond to metal binding is lower, resulting in a less stable complex. In the presence of K^+^ ions the complex stability seems to be ideally tuned, as the maximum catalytic activity of the enzyme is observed. The complex is stable enough to keep the enzyme in the closed state for the time required to complete the enzymatic reaction and, therefore, the catalytic cycle can be completed by changing the enzyme conformation from closed to open.

Ligand binding and enzymatic activity are impeded when other alkali cations are used. This could be explained by the lower stability of the SAHase_closed_-Ligand-Me^+^ complexes. Similar enzymatic activity and adenosine binding constants are observed in the presence of Na^+^ and Cs^+^ ions. The ionic radius of Cs^+^ (1.67 Å, coordination number VI) is larger than of other alkali ions and thus it should destabilize the complex. Another possible explanation is that Cs^+^, because of its size, does not fully occupy the available binding sites. The ionic radius of Na^+^ is significantly smaller (1.02 or 1.12 Å, coordination number VI or VII, respectively) than of K^+^. Therefore, the stability of the SAHase_closed_-Ligand-Na^+^ complex is lower, as the ionic contribution to Na^+^ coordination is higher than in complexes containing Rb^+^ or K^+^ ions. Additionally, numerous sodium-containing SAHase models indicate that the distance between the Na^+^ ion and the amide Oε atom of Q65 is too long and that this Na^+^–Q65 amide (which is still ordered) interaction should be considered as purely ionic; this would further reduce the stability of the complex. In consequence, complexes containing Cs^+^ or Na^+^ are not stable enough to support a high rate of the catalyzed reaction. Even lower (but comparable) activities are observed for the reactions carried out in the presence of Li^+^ (0.76 Å, coordination number VI) or in the absence of any alkali metal. Moreover, no heat effect was detected during the adenosine binding ITC experiment in in the presence of Li^+^. As proposed above, binding of the monovalent cation does not take place when the enzyme is in the open conformation, but occurs only after the conformational open-closed change that allows Q65 to approach the cation. Therefore, the possibility of the formation of an SAHase_closed_-Ligand-Li^+^ complex is questionable and the results may suggest that the Li^+^ ion is too small to be coordinated and, therefore, is unable to order the side chain of Q65.

### Zn^2+^ is a potent inhibitor of PaSAHase

**Figure 6:**
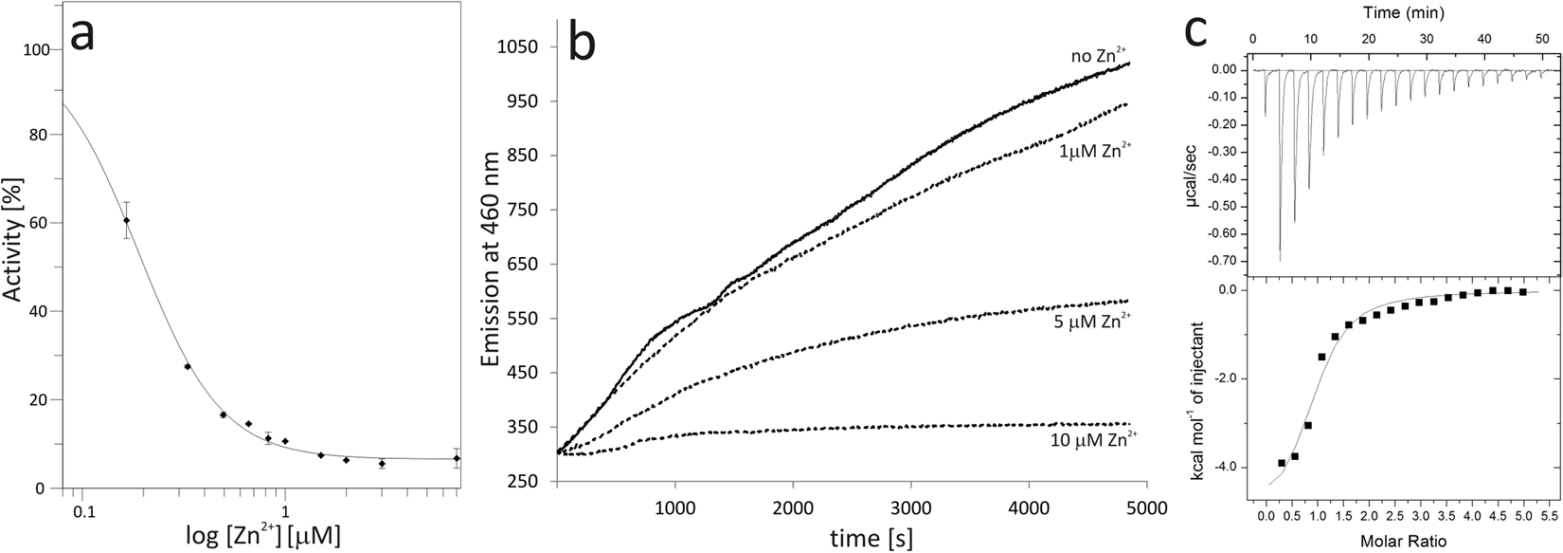
PaSAHase-Zn^2+^ interactions. (**a**) Inhibition of PaSAHase by Zn^2+^ ions. The wild-type Zn^2+^ form of the enzyme was incubated with different concentrations of Zn^2+^ ranging from 0.165 to 20 μM (shown on log scale). The enzymatic activity was followed in the hydrolytic direction by monitoring homocysteine formation *via* its reaction with 5,5′-dithiobis(2-nitrobenzoic acid). For a sample without Zn^2+^ the activity was 100%. (**b**) Time course of inactivation of PaSAHase by 2′-deoxyadenosine. PaSAHase (15 μM) was incubated with adenosine and Zn^2+^ or without the divalent cation. Irreversible formation of the reduced (and, therefore, inactive) form of the cofactor (NADH) was monitored spectrofluorimetrically after the addition of the competitive inhibitor 2′-deoxyadenosine. (**c**) Calorimetric titration (at 293 K) of PaSAHase with Zn^2+^ ions in the presence of 100 mM K^+^ ions. The top plot of the panel represents the raw heat data obtained from consecutive injections of a solution containing 2 mM Zn^2+^ ions into the sample cell containing 45 µM enzyme. The heat peak areas have been plotted against the molar ratio of Zn^2+^ ions added to the enzyme, creating the binding isotherm at the bottom of the panel. The best fit of the model of one set of binding sites is represented by the solid line.

The Zn^2+^ cation was incorporated into recombinant PaSAHase during expression and co-purifies with the enzyme. It was identified in two crystal structures of the enzyme and its presence was additionally confirmed by X-ray fluorescence spectroscopy (Supplementary Information Fig. S2). It should be noted that this is the first report of a divalent cation identification in any SAHase structure. Co-purification of Zn^2+^ with PaSAHase indicates that the enzyme has a very high affinity for this cation. First of all, the recombinant protein was purified using affinity chromatography with Ni^2+^ ions, which are chemically very similar to Zn^2+^. However, no nickel ions were detected in the protein sample using XRF. Secondly, although the total intracellular Zn^2+^ concentration is in the millimolar range, it is as low as pico- to femtomolar for free zinc ions^31–34^. In *E*. *coli* cells the concentration of free zinc ions has been established as ~20 pM^35^. Our enzymatic assays revealed that recombinant PaSAHase has only residual activity. Efficient removal of the Zn^2+^ ions (and of any metal ions for that matter) using TPEN restored the enzymatic activity, which shows that the zinc ions inhibit the activity of PaSAHase. Specifically, our kinetics studies based on PaSAHase inactivation (Fig. 6a) revealed that a tightly bound Zn^2+^ cation acts as a very strong noncompetitive inhibitor with an inhibitory constant K_i_ as low as 15 nM. Furthermore, the enzymatic study indicated that Zn^2+^ locks the enzyme-ligand complex in the closed conformation. This assay was performed analogously to the tests for the stability of the SAHase_closed_-Ligand-Me^+^ complexes that utilized 2′-deoxyadenosine. In the Zn^2+^ case, PaSAHase was first incubated in the presence of K^+^ ions with adenosine and Zn^2+^ ions at various concentrations, and subsequently, 2′-deoxyadenosine was added. The rate of NADH formation was monitored spectrofluorimetrically (Fig. 6b). The highest rate was observed for the reaction without any addition of Zn^2+^ and gradually decreased with increasing Zn^2+^ concentration. These results clearly indicate that the Zn^2+^ ions arrest the enzyme in the closed conformation stymieing the exchange of adenosine to 2′-deoxyadenosine.

Our crystal structures help to elucidate this puzzling behavior by showing that there is a tetrahedral zinc binding site, formed by three highly conserved amino acid residues involving S (Cys85), N (His323), and two O (Asp139) ligand atoms, located in the active site, where - together with the H323-F324 molecular gate - the divalent cation shuts access to the active site of the enzyme. Moreover, the Zn^2+^ cation is located at the interface between the two principal domains and is coordinated by three ligands from the substrate-binding domain (C85 Sγ, D139 O and D139 Oδ1) and one ligand from the cofactor-binding domain (H323 Nε1), thus further stabilizing the SAHase_closed_-Ligand-K^+^ complex. The zinc binding sites are not fully occupied and, therefore, the side chain of H323 from the molecular gate is modeled in two states, differing in ~180° flip of the imidazole ring. In both conformations the molecular gate remains shut. The rotation of the H323 side chain in the Zn^2+^ complex is necessary for the formation of the coordination bond. The second rotamer interacts *via* the H323 Nδ1 atom with the O5′ atom of adenosine. The dual conformation of H323 is correlated with a similar disorder of the adenosine O5′ atom. The details of the zinc ion coordination are included in Fig. 1a,b and Supplementary Information (Table S5**)**.

The mechanism of PaSAHase inactivation by zinc cations is different from that proposed for the inhibition of human placental SAHase activity by Cu^2+^ ions^17^. The latter study revealed that binding of copper cations affects protein-NAD^+^ interactions. In turn, the cofactor is released from the enzyme and the catalytic cycle cannot proceed anymore. The inhibition constants of zinc (~15 nM) and copper (~25 nM)^17^ ions are very low and similar. It was suggested that the copper-SAHase interactions may have biological significance, e.g., in the regulation of cellular methylation reactions or copper metabolism^36–40^. Contrary to that speculation, so far there is no literature evidences on any biological cross-talk between SAHase and zinc ions. However, since the total intracellular zinc concentration is in the range of millimolar, such an interaction with PaSAHase cannot be excluded.

PaSAHase-Zn^2+^ interactions were also tested by ITC experiments (Fig. 6c). Upon titration in the presence of K^+^ cations, the stoichiometry of protein:Zn^2+^ binding is around one zinc cation (0.90 ± 0.06) per one subunit with the apparent binding constant K_d_ of 12.7 ± 2.9 μM. The ITC results are in agreement with the crystallographic models of PaSAHase which also indicate the presence of only one Zn^2+^ site per subunit.

## Discussion

Monovalent cations are known to activate numerous enzymes but the mechanisms of their action could be different. Two types of activation have been proposed^41^. Activation is defined as type I for the enzymatic reactions where the cation interacts directly with a substrate to facilitate its binding (type Ia) or hydrolysis (type Ib). Frequently, a monovalent cation is utilized during the catalytic cycle in synergy with an additional divalent cation, e.g., Mg^2+^, Mn^2+^ or Zn^2+ ^^42–46^. A characteristic feature of enzymes activated by type I mechanism is a strong preference for a particular monovalent cation to ensure the highest catalytic activity^43,47–50^. This preference is usually related to a specific spatial arrangement of the active site or to the occurrence of optimal ionic interactions with a substrate provided by a particular monovalent cation^41^. On the other hand, the type of monovalent cation is not so strict for enzymes activated by type II mechanism. A striking feature of type II activation is that the monovalent cation does not interact with the substrate directly. The mechanism of type II activation is based on structural changes upon the monovalent cation binding, which could occur at various (e.g., local or/and global) level(s). Therefore, the detailed mechanism of this activation might be difficult to fully explain. For instance, binding of monovalent ion(s) could induce conformational changes within the whole subunit, the active site, or allosteric sites, and this in turn could facilitate substrate binding^51–58^. Additionally, other effects could also be observed, for instance, stabilization of the quaternary structure^53^, shifting of the equilibrium between the active and inactive conformers, or rigidifying of the enzyme structure and increasing protein thermal stability^57–59^.

*S*-adenosyl-l-homocysteine hydrolase obviously does not fall in the type I category as the monovalent cation does not interact with the ligand directly. It is true that the activity of PaSAHase depends on the cation type with a strong preference for K^+^, but the activation seems to be more similar to the situation defined for the type II mechanism. In SAHases, the monovalent cation participates in the ordering and proper orientation of Q65, the key residue involved in ligand binding. On the other hand, Q65 behaves similarly in the presence of different monovalent cations. Moreover, the conformation of the cation binding site in different SAHases is almost identical despite the presence of various monovalent cations, including NH_4_^+^, Na^+^, K^+^, or Rb^+^. Therefore, the strong preference of SAHases for K^+^ cannot be explained at the structural level alone. Our results strongly support the hypothesis that the role of the K^+^ ion is not restricted to fixing the orientation of the Q65 side chain. Instead, we propose that SAHases are activated by a special variant of type II mechanism, based on tuning of the enzyme dynamics (domain movement), which depends on the type of the coordinated cation. K^+^, but not other alkali cations, enables unique dynamic properties of the enzyme to ensure its maximum activity. The K^+^ ion stabilizes the enzyme-substrate complex in the closed conformation for a time interval required to complete the catalytic cycle. Stabilization of the closed state is achieved by (i) rigidifying the hinge region involved in substrate-induced domain reorientation and/or (ii) formation of additional polar contacts at the interface between the substrate- and cofactor-binding domains. The influence of metal cations on the enzyme dynamics can be exploited for enzyme inhibition. For example, the Rb^+^ cation is an inhibitor occupying the potassium site at the interdomain hinge. On the other hand, the potent inhibitory effect of Zn^2+^ is based on binding at the molecular gate and arresting the enzyme in the closed conformation.

## Methods

### Cloning, expression and purification of PaSAHase and Q65A mutein

The coding sequence of the *ahcY* gene was amplified by PCR from *P*. *aeruginosa* PAO1 genomic DNA. The amplicon was cloned into pMCSG57 expression vector using the ligation independent cloning reaction. The construct was used for Q65A mutant generation via PIPE^60^ cloning with the following primers: 5′-ATCGCGACCGGCGTGCTGATCGAGACCCTGGTC and 5′-GGTCGCGATGGTCATGTGGATGCAACCGAGGAT. The sequence of both constructs was confirmed by sequencing. The constructs carrying the coding sequence of the wild type PaSAHase or its Q65A mutant were transformed into BL21-CodonPlus(DE3)^®^-RIPL *E*. *coli* cells and expressed. 10 mL of LB medium containing 34 μg·mL^−1^ chloramphenicol and 100 μg·mL^−1^ ampicillin were inoculated and grown overnight at 310 K and the culture was used for inoculation of 1 L of LB medium, which was subsequently cultivated with appropriate antibiotics to an OD_600_ of 0.8. The temperature was decreased to 291 K and protein expression was induced with IPTG at a final concentration of 0.35 mM. The cells were harvested 20 hours after induction and flash-frozen in liquid nitrogen.

The purification procedure was the same for the wild type enzyme and Q65A mutein. The cell pellet was resuspended in buffer A (20 mM imidazole, 500 mM NaCl, 20 mM Tris·HCl pH 8.0, 10% glycerol) with the addition of 1 mM TCEP·HCl (to enhance enzyme stability) and 100 μg·mL^−1^ lysozyme. Cells were disrupted by sonication on ice and centrifuged for removal of debris. The supernatant was loaded onto a HisTrap column equilibrated with 0.05 M NiSO_4_. The protein was eluted with a buffer containing 300 mM imidazole, 500 mM NaCl, 20 mM Tris·HCl pH 8.0, 10% glycerol, and 1 mM TCEP·HCl. TEV protease was added at the final concentration of 0.1 mg·mL^−1^ and the protein solution was extensively dialyzed against buffer A. After overnight incubation at 277 K, the solution was loaded onto a HisTrap column equilibrated with 0.05 M NiSO_4_ and the protein was eluted with buffer A, subsequently exchanged for buffer B (100 mM KCl, 25 mM Tris·HCl pH 8.0, 1 mM TCEP·HCl) *via* dialysis. Next, a modified procedure of Yuan *et al*.^20^ was used for the preparation of the apo form of the enzyme. Briefly, a solution containing 5 mg of recombinant PaSAHase dissolved in 5 mL of buffer B was gradually mixed with 10 mL of saturated solution of (NH_4_)_2_SO_4_ at pH 3.3, and then stored for 10 min on ice. The mixture was centrifuged and the precipitate was dissolved in 5 mL of buffer C (100 mM KCl, 25 mM Tris·HCl pH 8.0). The enzyme was precipitated again as above and the pellet was washed with saturated neutral solution of (NH_4_)_2_SO_4_. Finally, the precipitated apo PaSAHase was dissolved in 2 mL of buffer C and subsequently NAD^+^ was added to a final concentration of 2 mM. After 30 minutes of incubation on ice, the mixture was loaded onto a Superdex 200 (Pharmacia) gel filtration column pre-equilibrated with buffer D (100 mM KCl, 25 mM HEPES·KOH pH 7.5, 1 mM TCEP·HCl). The protein was eluted with buffer D as a tetramer. Fractions with PaSAHase were concentrated to 20 mg·mL^−1^ by ultracentrifugation and the fresh protein solution was used for initial activity study, X-ray fluorescence experiments, and crystallization of Zn^2+^-containing PaSAHase complexes. For other studies, the purification procedure was modified by introduction of an additional step required to efficiently remove the Zn^2+^ ions by a strong chelator. Briefly, the gel filtration step was preceded with an extensive dialysis of the sample in buffer C supplemented with 50 μM *N*,*N*,*N′*,*N′-*tetrakis(2-pyridinylmethyl)−1,2-ethanediamine (TPEN). The subsequent purification steps were unchanged.

Both purified proteins are extended at the N-terminus by a short tripeptide (SNA-) cloning artifact. SDS-PAGE analysis confirmed the size of the expressed proteins (~53 kDa). Finally, mass spectrometry was used to confirm the size and sequence of the wild type enzyme.

### Assays for SAHase activity and inhibition

Assays for PaSAHase activity in the hydrolytic direction were carried out spectrophotometrically and the rate of L-homocysteine (Hcy) formation was measured by monitoring its reaction with 5,5′-dithiobis(2-nitrobenzoic acid) (DTNB)^21^. Assays were performed in 3 mL volume in a buffer containing 100 mM MeCl (where Me^+^ corresponds to Li^+^, Na^+^, K^+^, Rb^+^, or Cs^+^ ion) and 25 mM HEPES·MeOH pH 7.5, or a buffer containing 100 mM Tris·HCl pH 7.5. For the Q65A mutein, the experiments were conducted in a buffer containing 100 mM KCl and 25 mM HEPES·KOH pH 7.5. The buffers were supplemented with six units of adenosine deaminase, 100 μM DTNB, and 0.32 μM of PaSAHase or its mutein. The reaction was initiated by the addition of *S*-adenosyl-l-homocysteine (SAH) at a final concentration from 2.5 to 200 μM. The conversion of Hcy to Hcy-TNB was carried out at 293 K. The reaction progress was measured for one minute and monitored at 412 nm using a U-3900H spectrophotometer (Hitachi). Initial velocity parameters were estimated from the linear region of the recorded curve.

For the inhibition study, PaSAHase (0.35 μM) in a buffer containing 100 mM KCl and 25 mM Tris·HCl pH 7.5 was incubated with Zn^2+^ at different concentrations, ranging from 0.165 to 20 μM. The reaction was initiated by the addition of SAH at a final concentration of 85 μM. The enzymatic activity was followed in the hydrolytic direction by monitoring homocysteine formation *via* its reaction with DNTB to determine the IC_50_ constant. For the sample without the addition of Zn^2+^ ions, 100% activity was assigned. All measurements were performed in duplicates. The experimental data were analyzed with the *GraFit* 7.0 software (Erithacus) to obtain the kinetic parameters of the enzymatic reactions: substrate affinity (K_M_), activity (V_max_), as well as catalytic rate (k_cat_) or IC_50_ value for inhibition. The IC_50_-to-K_i_ converter^61^ was applied to calculate the inhibition constant (K_i_) for Zn^2+^ ions using the noncompetitive model of inhibition.

### Isothermal titration calorimetry

ITC measurements were performed at 293 K using a MicroCal iTC200 calorimeter (GE Healthcare). For the adenosine binding studies, the wild type protein was dialyzed against a buffer containing 100 mM MeCl and 25 mM HEPES·MeOH pH 7.5, or an alkali-cation-free buffer containing 100 mM Tris·HCl pH 7.5. The experiment for the Q65A mutein was conducted in a buffer containing 100 mM KCl and 25 mM HEPES·KOH pH 7.5. The final protein concentrations were in the range of 100–120 µM. Adenosine was dissolved in the dialysis buffers to the concentrations of 0.6 to 1.0 mM. The K^+^ ion binding study was conducted in 50 mM Tris·HCl pH 7.5 at PaSAHase concentration of 70 μM. The protein was titrated with 1 mM KCl in 50 mM Tris·HCl pH 7.5. The Zn^2+^ ion binding study was conducted in a buffer containing 100 mM KCl and 50 mM Tris·HCl pH 7.5 at PaSAHase concentration of 45 μM. The protein was titrated with 2 mM ZnCl_2_. In all ITC experiments the ligand was injected to the protein solution (300 μL) in 2 µL aliquots until saturation was observed. The protein concentrations were estimated by the Bradford method and are presented for a single PaSAHase subunit. The raw ITC data were analyzed with the *ORIGIN* 7.0 software (OriginLab) to obtain the thermodynamic parameters of the complexation reactions: stoichiometry (N), dissociation constant (K_d_), as well as changes in enthalpy (ΔH) and entropy (ΔS). For sigmoidal titration curves, the *one set of sites* model was fitted and N was obtained from the experiment. For hyperbolic curves, for which the determination of N is impossible, N was fixed while fitting the same model. All measurements were performed in duplicates.

### Time course of PaSAHase inactivation by 2′-deoxyadenosine

The measurements were performed at 293 K using an F-7000 spectrofluorotometer (Hitachi) in the volume of 1 mL. Irreversible formation of the reduced form of the cofactor (NADH) was monitored spectrofluorometrically by excitation at 340 nm and measurement of emission at 460 nm, as described previously^20^. The influence of K^+^ and Rb^+^ ions on the time-dependent inactivation of the enzyme was carried out for 15 μM PaSAHase in a buffer containing 100 mM KCl or RbCl, 25 mM HEPES·KOH or HEPES·RbOH pH 7.5, and 1 mM TCEP·HCl. In the first experiment, the reaction was initiated by the addition of 2′-deoxyadenosine to the final concentration of 5 mM. In the second experiment, the enzyme was incubated for 2 hours with 100 μM adenosine and subsequently 2′-deoxyadenosine was added at a final concentration of 200 μM. After one minute the reaction was monitored for 4000 seconds (first experiment) or until the intensity of fluorescence emission reached a plateau (second experiment). An analogous experiment was carried out to analyze the influence of Zn^2+^ cations on the enzyme. Herein, 15 μM PaSAHase in a buffer containing 100 mM KCl and 25 mM HEPES·KOH was incubated for 2 hours with adenosine (200 μM) and ZnCl_2_ (1, 5 or 10 μM) or without the divalent cation. The cofactor conversion reaction was initiated by the addition of 2′-deoxyadenosine to the final concentration of 5 mM and after one minute was monitored for 5000 seconds.

### X-ray fluorescence spectroscopy

Freshly purified PaSAHase in buffer C from the batch not treated with Zn^2+^ chelator was suspended in a cryoloop with the diameter of 1.0 mm and vitrified in liquid nitrogen prior to X-ray fluorescence detection. Initial X-ray fluorescence spectrum for the energy range from 8000 to 11500 eV was recorded at BESSY beamline 14.1 using Amptek X-123 SDD X-ray spectrometer. Maximum of emission related to the presence of zinc ions was observed in the energy range from 9600–9700 eV (Supplementary Fig. 2).

### ^23^Na NMR Spectroscopy

^23^Na NMR spectra were measured on a BRUKER Avance 500 MHz spectrometer (5 mm BBFO BB-^19^F/^1^H Z-gradient probe). Six samples were prepared in 50 mM Tris·HCl pH 7.5, which contained: (1) 5 mM NaCl, (2) 5 mM NaCl + 1 mM Ado, (3) 5 mM NaCl + 0.3 mM PaSAHase, (4) 0.3 mM PaSAHase + 5 mM NaCl + 1 mM Ado, (5) 0.3 mM PaSAHase + 0.6 mM NaCl + 1 mM Ado, (6) 0.3 mM PaSAHase + 0.3 mM NaCl + 1 mM Ado. ^23^Na NMR chemical shifts are given in ppm relative to 5 mM NaCl in 50 mM Tris·HCl pH 7.5.

### Crystallization and X-ray data collection

Protein solution in a buffer containing 100 mM KCl or RbCl, 25 mM HEPES·KOH or HEPES·RbOH pH 7.5, and 1 mM TCEP·HCl was incubated overnight with 2 mM of adenosine (Ado/K^+^/Zn^2+^), 2′-deoxyadenosine (2′-dAdo/K^+^/Zn^2+^ or 2′-dAdo/Rb^+^), or 3′-deoxyadenosine (3′-dAdo/K^+^) at 277 K. Crystallization drops were mixed from 2 μL of the protein solution (10 mg∙mL^-1^ measured spectrofotometrically at 280 nm) and 2 μL of the reservoir solution containing 20% (w/v) PEG8000 and 50 mM KH_2_PO_4_ (Ado/K^+^/Zn^2+^ and 2′-dAdo/K^+^/Zn^2+^), or 20% (w/v) PEG8000 and 50 mM RbH_2_PO_4_ (2′-dAdo/Rb^+^), or 15% (w/v) PEG8000, 50 mM KH_2_PO_4_ and 20% (v/v) glycerol (3′-dAdo/K^+^). Diffraction-quality crystals were grown within a few days at 292 K using the hanging-drop vapor-diffusion method. X-Ray diffraction data were measured at BESSY beamlines 14.1, 14.2, 14.3 or APS beamline SER-CAT 22-ID. Crystals of the 3′-dAdo/K^+^ complex were fished out directly from mother liquor and vitrified in liquid nitrogen. Other crystals were soaked for a few seconds in the reservoir solution supplemented with 20% (v/v) glycerol and vitrified in liquid nitrogen prior to X-ray data collection. The diffraction data extend to 1.35 Å (3′-dAdo/K^+^), 1.45 Å (2′-dAdo/Rb^+^), 1.60 Å (Ado/K^+^/Zn^2+^), or 1.75 Å (2′-dAdo/K^+^/Zn^2+^) resolution. Indexing and integration of the diffraction images was performed in the *HKL3000* package^62^ (2′-dAdo/K^+^/Zn^2+^ and 3′-dAdo/K^+^) or *XDSAPP* graphical interface^63^ of the *XDS* package^64^ (Ado/K^+^/Zn^2+^ and 2′-dAdo/Rb^+^). The intensity data were scaled in *HKL3000* or *XDS*. The final data sets are characterized in Supplementary Table S1.

### Structure solution, refinement and validation

The crystal structure of PaSAHase in the 3′-dAdo/K^+^ complex was solved by molecular replacement, as implemented in the program *PHASER*^65^ from the *CCP4* suite^66^ using a subunit of *H*. *sapiens* SAHase^27^ as the search model (PDB entry 1LI4, chain A). The correct solution was found in space group *C*2 with two subunits in the asymmetric unit, from which crystallographic symmetry generates the complete PaSAHase homotetramer. The other three crystal structures were solved by molecular replacement using *PHASER* and a subunit from the 3′-dAdo/K^+^ solution as a search probe. The correct solutions were found in space group *C*2 with four subunits in the asymmetric unit forming the PaSAHase tetramer. For all four complexes, automatic model building was carried out with the online version of *ARP/wARP*^67^. Anisotropic (3′-dAdo/K^+^, 2′-dAdo/Rb^+^) or isotropic (Ado/K^+^/Zn^2+^, 2′-dAdo/K^+^/Zn^2+^) stereochemically-restrained structure-factor refinement was carried out in *REFMAC5*^68^ with maximum-likelihood targets and with the inclusion of three TLS groups^69^ (Ado/K^+^/Zn^2+^ and 2′-dAdo/K^+^/Zn^2+^) per protein chain, as suggested by the *TLSMD* server^70^. The ligands were identified without ambiguity in *mF*_*o*_*-DF*_*c*_ omit electron density maps phased with the contribution of the protein atoms only. The occupancy of the zinc ions was adjusted manually to satisfy simultaneously two conditions: (i) clean difference maps and (ii) ADP parameters of the zinc ions similar to those of the adjacent atoms. The alkali cation binding sites were confirmed with *CheckMyMetal*^71^. Additionally, the Rb^+^ ions were clearly present in anomalous difference Fourier maps. The *COOT* program^72^ was used for manual modeling in electron density maps. Stereochemical quality of the models was assessed using the wwPDB validation pipeline^73^. Final refinement statistics for all four crystal structures are reported in Supplementary Table 1.

### Other software used

The *SSM* algorithm^74^ was used for Cα superposition of protein models. Molecular figures were generated in *PyMOL*^75^. The multipole sequence alignment was calculated in *Clustal Omega*^76^.

### Data availability

Atomic coordinates and ADP parameters, as well as structure factors have been deposited in the Protein Data Bank (PDB) with the accession codes 6F3M (Ado/K^+^/Zn^2+^), 6F3O (2′-dAdo/K^+^/Zn^2+^), 6F3P (3′-dAdo/K^+^), and 6F3Q (2′-dAdo/Rb^+^). Raw diffraction images were deposited in the Integrated Resource for Reproducibility in Macromolecular Crystallography (ProteinDiffraction.org) with the following DOIs: https://doi.org/10.18430/M36F3M (Ado/K^+^/Zn^2+^), https://doi.org/10.18430/M36F3O (2’-dAdo/K^+^/Zn^2+^), https://doi.org/10.18430/M36F3P (3’-dAdo/K^+^) and https://doi.org/10.18430/M36F3Q (2’-dAdo/Rb^+^).

## Electronic supplementary material

Supplementary Information

## References

[1] Richards HH, Chiang PK, Cantoni GL (1978). Adenosylhomocysteine hydrolase. Crystallization of the purified enzyme and its properties. J Biol Chem.

[2] Poulton JE, Butt VS (1975). Purification and properties of S-adenosyl-l-methionine: caffeic acid *O*-methyltransferase from leaves of spinach beet (*Beta vulgaris* L.). Biochim Biophys Acta.

[3] Chiang PK, Cantoni GL (1979). Perturbation of biochemical transmethylations by 3-deazaadenosine *in vivo*. Biochem Pharmacol.

[4] Chiang PK (1998). Biological effects of inhibitors of *S*-Adenosylhomocysteine hydrolase. Pharmacol Ther.

[5] Hershfield MS (1979). Apparent suicide inactivation of human lymphoblast *S*-adenosylhomocysteine hydrolase by 2′-deoxyadenosine and adenine arabinoside. A basis for direct toxic effects of analogs of adenosine. J Biol Chem.

[6] Brzezinski K, Dauter Z, Jaskolski M (2012). High-resolution structures of complexes of plant S-adenosyl-l-homocysteine hydrolase (*Lupinus luteus*). Acta Crystallogr D Biol Crystallogr.

[7] Turner MA (1998). Structure determination of selenomethionyl *S*-adenosylhomocysteine hydrolase using data at a single wavelength. Nat Struct Biol.

[8] Hu Y (1999). Crystal structure of *S*-adenosylhomocysteine hydrolase from rat liver. Biochemistry.

[9] Tanaka N (2004). Crystal structure of S-adenosyl-l-homocysteine hydrolase from the human malaria parasite *Plasmodium falciparum*. J Mol Biol.

[10] Reddy MCM (2008). Crystal structures of *Mycobacterium tuberculosis* S-adenosyl-l-homocysteine hydrolase in ternary complex with substrate and inhibitors. Protein Sci.

[11] Manszewski T, Singh K, Imiolczyk B, Jaskolski M (2015). An enzyme captured in two conformational states: crystal structure of *S* -adenosyl- L -homocysteine hydrolase from *Bradyrhizobium elkanii*. Acta Crystallogr D Biol Crystallogr.

[12] Zheng Y (2015). Crystal structures of *S*-adenosylhomocysteine hydrolase from the thermophilic bacterium *Thermotoga maritima*. J Struct Biol.

[13] Brzezinski K (2017). S-adenosyl-l-homocysteine hydrolase from a hyperthermophile (*Thermotoga maritima*) is expressed in *Escherichia coli* in inactive form - Biochemical and structural studies. Int J Biol Macromol.

[14] Kusakabe Y (2015). Structural insights into the reaction mechanism of S-adenosyl-l-homocysteine hydrolase. Sci Rep.

[15] Czyrko, J., Jaskolski, M. & Brzezinski, K. Crystal Structure of S-adenosyl-l-homocysteine hydrolase from *Cytophaga hutchinsonii*, a case of combination of crystallographic and noncrystallographic symmetry. *Croat Chem Acta* **91** (2018).

[16] Palmer JL, Abeles RH (1979). The mechanism of action of *S*-adenosylhomocysteinase. J Biol Chem.

[17] Li M (2007). Copper ions inhibit S-adenosylhomocysteine hydrolase by causing dissociation of NAD^+^ cofactor. Biochemistry.

[18] Komoto J (2000). Effects of site-directed mutagenesis on structure and function of recombinant rat liver *S*-adenosylhomocysteine hydrolase. Crystal structure of D244E mutant enzyme. J Biol Chem.

[19] Manszewski T, Szpotkowski K, Jaskolski M (2017). Crystallographic and SAXS studies of S-adenosyl-l-homocysteine hydrolase from *Bradyrhizobium elkanii*. IUCrJ.

[20] Yuan CS, Yeh J, Liu S, Borchardt RT (1993). Mechanism of inactivation of *S*-adenosylhomocysteine hydrolase by (Z)−4′,5′-didehydro-5′-deoxy-5′-fluoroadenosine. J Biol Chem.

[21] Yuan CS, Ault-Riché DB, Borchardt RT (1996). Chemical modification and site-directed mutagenesis of cysteine residues in human placental *S*-adenosylhomocysteine hydrolase. J Biol Chem.

[22] Lozada-Ramírez JD, Martínez-Martínez I, Sánchez-Ferrer A, García-Carmona F (2006). A colorimetric assay for *S*-adenosylhomocysteine hydrolase. J Biochem Biophys Meth.

[23] Turner MA (2000). Structure and function of *S*-adenosylhomocysteine hydrolase. Cell Biochem Biophys.

[24] Wang M (2006). Effects of Ligand Binding and Oxidation on Hinge-Bending Motions in S-adenosyl-l-homocysteine Hydrolase. Biochemistry.

[25] Clausen MJV, Poulsen H (2013). Sodium/Potassium Homeostasis in the Cell. Met Ions Life Sci.

[26] Stepkowski T, Brzeziński K, Legocki AB, Jaskólski M, Béna G (2005). Bayesian phylogenetic analysis reveals two-domain topology of *S*-adenosylhomocysteine hydrolase protein sequences. Mol Phylogenet Evol.

[27] Yang X (2003). Catalytic strategy of S-adenosyl-l-homocysteine hydrolase: transition-state stabilization and the avoidance of abortive reactions. Biochemistry.

[28] Yin D (2000). Substrate binding stabilizes *S*-adenosylhomocysteine hydrolase in a closed conformation. Biochemistry.

[29] Schomburg I (2017). The BRENDA enzyme information system–From a database to an expert system. J. Biotechnol..

[30] Shannon RD (1976). Revised effective ionic radii and systematic studies of interatomic distances in halides and chalcogenides. Acta Crystallogr Sect A.

[31] Outten CE (2001). Femtomolar Sensitivity of Metalloregulatory Proteins Controlling Zinc Homeostasis. Science.

[32] Jacobsen FE, Kazmierczak KM, Lisher JP, Winkler ME, Giedroc DP (2011). Interplay between manganese and zinc homeostasis in the human pathogen *Streptococcus pneumoniae*. Met Integr Biometal Sci.

[33] Begg, S. L. *et al*. Dysregulation of transition metal ion homeostasis is the molecular basis for cadmium toxicity in *Streptococcus pneumoniae*. *Nat Commun* **6** (2015). 10.1038/ncomms7418 PMC4366526 25731976

[34] Capdevila DA, Wang J, Giedroc DP (2016). Bacterial Strategies to Maintain Zinc Metallostasis at the Host-Pathogen Interface. J Biol Chem.

[35] Patzer SI, Hantke K (1998). The ZnuABC high-affinity zinc uptake system and its regulator Zur in *Escherichia coli*. Mol Microbiol.

[36] Bethin KE, Petrovic N, Ettinger MJ (1995). Identification of a major hepatic copper binding protein as *S*-adenosylhomocysteine hydrolase. J Biol Chem.

[37] McArdle HJ, Bingham MJ, Summer K, Ong TJ (1999). Cu metabolism in the liver. Adv Exp Med Biol.

[38] Mansoor MA (2000). Correlation between plasma total homocysteine and copper in patients with peripheral vascular disease. Clin Chem.

[39] Chen J, Liu Q (2002). Copper ion’ inhibition on activity of S-Adenosylhomocysteine hydrolase. Chin Sci Bull.

[40] Li Y, Chen J, Liu J, Yang X, Wang K (2004). Binding of Cu^2+^ to S-adenosyl-l-homocysteine hydrolase. J Inorg Biochem.

[41] Gohara DW, Di Cera E (2016). Molecular Mechanisms of Enzyme Activation by Monovalent Cations. J Biol Chem.

[42] Larsen TM, Laughlin LT, Holden HM, Rayment I, Reed GH (1994). Structure of rabbit muscle pyruvate kinase complexed with Mn^2+^, K^+^, and pyruvate. Biochemistry.

[43] Viitanen PV (1990). Chaperonin-facilitated refolding of ribulosebisphosphate carboxylase and ATP hydrolysis by chaperonin 60 (groEL) are K^+^ dependent. Biochemistry.

[44] Villeret V, Huang S, Fromm HJ, Lipscomb WN (1995). Crystallographic evidence for the action of potassium, thallium, and lithium ions on fructose-1,6-bisphosphatase. Proc Natl Acad Sci USA.

[45] Komoto J, Yamada T, Takata Y, Markham GD, Takusagawa F (2004). Crystal structure of the *S*-adenosylmethionine synthetase ternary complex: a novel catalytic mechanism of *S*-adenosylmethionine synthesis from ATP and Met. Biochemistry.

[46] Wu Y, He Y, Moya IA, Qian X, Luo Y (2004). Crystal structure of archaeal recombinase RADA: a snapshot of its extended conformation. Mol Cell.

[47] Toraya T, Sugimoto Y, Tamao Y, Shimizu S, Fukui S (1971). Propanediol dehydratase system. Role of monovalent cations in binding of vitamin B12 coenzyme or its analogs to apoenzyme. Biochemistry.

[48] Wheatley RW, Juers DH, Lev BB, Huber RE, Noskov SY (2015). Elucidating factors important for monovalent cation selectivity in enzymes: *E*. *coli* β-galactosidase as a model. Phys Chem Chem Phys.

[49] Marcus F, Hosey MM (1980). Purification and properties of liver fructose 1,6-bisphosphatase from C57BL/KsJ normal and diabetic mice. J Biol Chem.

[50] Wu Y, Qian X, He Y, Moya IA, Luo Y (2005). Crystal structure of an ATPase-active form of Rad51 homolog from *Methanococcus voltae*. Insights into potassium dependence. J Biol Chem.

[51] Paul R, Patra MD, Sen U (2015). Crystal structure of apo and ligand bound V*ibrio cholerae* ribokinase (Vc-RK): role of monovalent cation induced activation and structural flexibility in sugar phosphorylation. Adv Exp Med Biol.

[52] Hohenester E, Keller JW, Jansonius JN (1994). An alkali metal ion size-dependent switch in the active site structure of dialkylglycine decarboxylase. Biochemistry.

[53] AEvarsson A (2000). Crystal structure of human branched-chain alpha-ketoacid dehydrogenase and the molecular basis of multienzyme complex deficiency in maple syrup urine disease. Structure.

[54] Woehl EU, Dunn MF (1995). Monovalent metal ions play an essential role in catalysis and intersubunit communication in the tryptophan synthase bienzyme complex. Biochemistry.

[55] Peracchi A, Mozzarelli A, Rossi GL (1995). Monovalent cations affect dynamic and functional properties of the tryptophan synthase alpha 2 beta 2 complex. Biochemistry.

[56] Dunn MF (2012). Allosteric regulation of substrate channeling and catalysis in the tryptophan synthase bienzyme complex. Arch Biochem Biophys.

[57] Vogt AD, Bah A, Di Cera E (2010). Evidence of the E*-E equilibrium from rapid kinetics of Na^+^ binding to activated protein C and factor Xa. J Phys Chem B.

[58] Vogt AD, Chakraborty P, Di Cera E (2015). Kinetic dissection of the pre-existing conformational equilibrium in the trypsin fold. J Biol Chem.

[59] Lechtenberg BC, Johnson DJD, Freund SMV, Huntington JA (2010). NMR resonance assignments of thrombin reveal the conformational and dynamic effects of ligation. Proc Nat. Acad Sci USA.

[60] Klock HE, Koesema EJ, Knuth MW, Lesley SA (2008). Combining the polymerase incomplete primer extension method for cloning and mutagenesis with microscreening to accelerate structural genomics efforts. Proteins.

[61] Cer RZ, Mudunuri U, Stephens R, Lebeda FJ (2009). *IC*_50_*-to-K*_*i*_: a web-based tool for converting IC50 to Ki values for inhibitors of enzyme activity and ligand binding. Nucleic Acids Res.

[62] Otwinowski, Z. & Minor, W. Processing of X-ray diffraction data collected in oscillation mode. *Methods Enzymol* **276**, 307–326. 10.1016/S0076-6879(97)76066-X 27754618

[63] Sparta KM, Krug M, Heinemann U, Mueller U, Weiss MS (2016). *XDSAPP2*.*0*. J Appl Crystallogr.

[64] Kabsch W (2010). *XDS*. Acta Crystallogr D Biol Crystallogr.

[65] McCoy AJ (2007). *Phaser* crystallographic software. J Appl Crystallogr.

[66] Winn MD (2011). Overview of the CCP4 suite and current developments. Acta Crystallogr D Biol Crystallogr.

[67] Langer G, Cohen SX, Lamzin VS, Perrakis A (2008). Automated macromolecular model building for X-ray crystallography using ARP/wARP version 7. Nat Protoc.

[68] Murshudov GN (2011). *REFMAC* 5 for the refinement of macromolecular crystal structures. Acta Crystallogr D Biol Crystallogr.

[69] Winn MD, Isupov MN, Murshudov GN (2001). Use of TLS parameters to model anisotropic displacements in macromolecular refinement. Acta Crystallogr D Biol Crystallogr.

[70] Painter J, Merritt EA (2006). *TLSMD* web server for the generation of multi-group TLS models. J Appl Crystallogr.

[71] Zheng H (2017). *CheckMyMetal*: a macromolecular metal-binding validation tool. Acta Crystallogr D Struct Biol.

[72] Emsley P, Lohkamp B, Scott WG, Cowtan K (2010). Features and development of *Coot*. Acta Crystallogr D Biol Crystallogr.

[73] Berman H, Henrick K, Nakamura H (2003). Announcing the worldwide Protein Data Bank. Nat Struct Biol.

[74] Krissinel E, Henrick K (2004). Secondary-structure matching (SSM), a new tool for fast protein structure alignment in three dimensions. Acta Crystallogr D Biol Crystallogr.

[75] Schrödinger, LLC. The PyMOL Molecular Graphics System. *Version* 1.8. (2015).

[76] Sievers F (2011). Fast, scalable generation of high-quality protein multiple sequence alignments using Clustal Omega. Mol Syst Biol.

