# Supplementary material for: Metal-cation regulation of enzyme dynamics is a key factor influencing the activity of S-adenosyl-l-homocysteine hydrolase from Pseudomonas aeruginosa
Source: Sci Rep. 2018 Jul 27;8:11334. doi: 10.1038/s41598-018-29535-y (PMC6063907; doi:10.1038/s41598-018-29535-y)
Supplement: Supplementary file 1 — Supplementary Information [file 41598_2018_29535_MOESM1_ESM.pdf]

Electronic Supplementary Information for

Metal-cation regulation of enzyme dynamics is a key factor influencing the activity of *S*-adenosyl-L-homocysteine hydrolase from *Pseudomonas aeruginosa*

Justyna Czyrko[1], Joanna Sliwiak[2], Barbara Imiolczyk[2], Zofia Gdaniec[3], Mariusz Jaskolski[2,4], & Krzysztof Brzezinski[1*]

[1] Laboratory of Biochemistry and Structural Biology, Institute of Chemistry, University of Bialystok, Poland

[2] Center for Biocrystallographic Research, Institute of Bioorganic Chemistry, Polish Academy of Sciences, Poznan, Poland

[3] Laboratory of Biomolecular NMR, Institute of Bioorganic Chemistry, Polish Academy of Sciences, Poznan, Poland

[4] Department of Crystallography, Faculty of Chemistry, A. Mickiewicz University, Poznan, Poland

*To whom correspondence should be addressed,
Email: k.brzezinski@uwb.edu.pl

Table of Contents

Tables..3-5
Figures..6-7

Table S1. Crystallographic data, data collection and structure refinement statistics. Values in parentheses are for the last resolution shell.

Added ligand/present cation(s)	Ado/K^+/Zn^{2+}	2'-dAdo/K^+/Zn^{2+}	3'-dAdo/K^+	2'-dAdo/Rb^+
Data collection				
Beamline	BESSY 14.2	BESSY 14.3	APS 22-ID	BESSY 14.1
Wavelength (A˚)	0.9184	0.8950	1.0000	0.8077
Temperature (K)	100	100	100	100
Space group	*C*2	*C*2	*C*2	*C*2
Unit-cell parameters (Å,°)				
a	175.98	170.84	142.90	170.49
b	104.14	99.61	85.74	99.44
c	107.63	111.82	112.01	111.71
β	100.7	102.0	122.2	101.9
Resolution (Å)	50.0 – 1.60 (1.69 – 1.60)	25.0 – 1.75 (1.81 – 1.75)	30.0 – 1.35 (1.40 – 1.35)	50.0 – 1.45 (1.54 – 1.45)
Mosaicity (°)	0.1	0.4	0.6	0.1
Completeness (%)	97.8 (90.0)	99.9 (99.5)	98.6 (95.8)	98.6 (97.7)
Multiplicity	3.2 (2.8)	4.2 (4.2)	2.9 (2.6)	4.0 (4.0)
$\langle I/\sigma(I) \rangle$	14.9 (3.0)	17.6 (3.2)	13.1 (2.0)	16.6 (3.3)
R_{merge}†	0.061 (0.403)	0.078 (0.415)	0.066 (0.481)	0.049 (0.340)
Refinement statistics				
Working/test reflections	242459/2450	181035/1806	244097/1220	315012/3343
R/R_{free}‡	0.151/0.174	0.140/0.167	0.101/0.127	0.093/0.124
No. of atoms				
Protein	14335	14445	7401	14554
Ligand	76	40	36	40
NAD^+	176	176	88	176
Water	2021	1632	1132	2166
Phosphate ions	4	8	2	8
Cl^- ions	-	-	2	-
K^+ ions	4	4	2	-
Zn^{2+} ions	4	2	-	-
Rb^+ ions	-	-	-	5
Glycerol molecules	3	1	-	1
PEG molecules	-	-	-	1
R.m.s.d. from ideality				
bond lengths (Å)	0.013	0.015	0.016	0.018
bond angles (°)	1.58	1.66	1.77	1.80
Average B factor (Å^2)	27.0	26.0	19.0	17.0
Ramachandran statistics (%)				
Most favored regions	98	98	98	98
Allowed regions	2	2	2	2
PDB code	6F3M	6F3O	6F3P	6F3Q

Table S2. Polar interactions with the ligand molecules bound in active site of PaSAHase, with the corresponding donor-acceptor distances (Å) in parentheses. Amino acid residues from the substrate (*) and cofactor (#) binding domains, as well as the monovalent cation coordinating loop ($) are involved in ligand binding. The interactions in all four subunits are almost identical; therefore, the distances are only listed for subunit A.

Protein/Phosphate atom	Ado/K^+/Zn^{2+} Adenosine atom	2'-dAdo/K^+/Zn^{2+} Adenine/Phosphate atom	2'-dAdo/Rb^+ Adenine/Phosphate atom	3'-dAdo/K^+ Cordycepin atom
*T63 Oγ	N1 (2.75)	N1 (2.74)	N1 (2.69)	N1 (2.73)
*Q65 Oε	N6 (2.85)	N6 (2.84)	N6 (2.87)	N6 (2.87)
$H382 O	N6 (3.11)	N6 (3.12)	N6 (3.04)	N6 (3.09)
$H382 N	N7 (2.95)	N7 (2.89)	N7 (2.93)	N7 (2.94)
Phosphate O2	-	N9 (3.18)	N9 (3.16)	-
Phosphate O4	-	N9 (2.96)	N9 (2.93)	-
*E164 Oε2	O2' (2.67)	O3 (2.59)	O3 (2.49)	O2' (2.61)
#D198 Oδ1	-	O2 (2.87)	O2 (2.76)	-
#D198 Oδ2	O2' (2.50)	O3 (2.65)	O3 (2.49)	O2' (2.56)
*T165 Oγ	O3' (2.70)	O1 (2.72)	O1 (2.79)	-
#K194 Nζ	O3' (2.78)	O1 (2.78)	O1 (2.68)	-
*H61 Nε2	O5' (2.77)	-	-	O5' (2.90)
*D139 Oδ1	O5' (2.76)	-	-	O5' (2.66)
*D139 Oδ2	-	O4 (2.71)	O4 (2.62)	-
#H323 Nδ1	O5' (2.68)	-	-	O5' (2.63)

Table S3. Details of alkali cation coordination near the active site of PaSAHase in all four complex structures with the corresponding cation-ligand distances in Å. The distances are listed for all chains (A-D).

Ligand atom:	Q65 Oε1	T380 O	T380 Oγ	H382 O	Wat1	Wat2	Wat3
Ado/K^+/Zn^{2+}							
A	3.21	2.71	2.83	2.74	2.74	3.09	2.82
B	3.18	2.68	2.90	2.74	2.75	3.06	2.80
C	3.20	2.70	2.86	2.73	2.78	3.06	2.84
D	3.19	2.68	2.83	2.76	2.80	3.08	2.76
2'-dAdo/K^+/Zn^{2+}							
A	3.20	2.70	2.80	2.78	2.81	2.95	2.86
B	3.18	2.62	2.85	2.81	2.82	3.01	2.83
C	3.17	2.65	2.88	2.80	2.76	3.07	2.83
D	3.19	2.61	2.89	2.77	2.80	2.96	2.76
3'-dAdo/K^+							
A	3.20	2.72	2.84	2.75	2.74	3.08	2.78
C	3.17	2.71	2.84	2.73	2.76	3.09	2.81
2'-dAdo/Rb^+							
A	3.22	2.84	2.88	2.87	2.94	3.12	3.01
B	3.20	2.84	2.90	2.88	2.90	3.18	2.97
C	3.21	2.84	2.88	2.88	2.92	3.14	2.99
D	3.22	2.83	2.91	2.89	2.92	3.14	2.98

Table S4. Comparison of the monovalent cation interactions with the anchoring glutamine or glutamate residue near the active site of selected SAHase models in the closed conformation.

Enzyme source	PDB ID	approaching residue	cation	type of interaction
P. aeruginosa	this work	Q65	K^+	direct
B. melitensis	3N58	Q59	K^+	direct
B. elkani	4LVC	Q62	NH_4^+	*via* water molecule
B. elkani	5M5K	Q62	Na^+	direct
M. tuberculosis	3CE6	Q73	Na^+	*via* water molecule
B. pseudomallei	3GLQ	Q66	Na^+	direct
L. luteus	3OND	Q66	Na^+	*via* water molecule
H. sapiens	1LI4	E59	Na^+ or NH_4^+	direct
P. falciparum	1V8B	E58	Na^+	*via* water molecule
N. fowleri	5V96	E63	Na^+	direct

Table S5. Details of zinc coordination in two complex structures, with the corresponding ligand-Zn^{2+} distances in Å. The interactions are listed for all four (A-D) subunits.

Ligand atom:	C85 Sγ	D139 O	D139 Oδ1	H323 Nε1	Zn^{2+} occupancy
**Ado/K^+/Zn^{2+}**					
A	2.32	2.22	2.11	2.07	0.5
B	2.35	2.30	2.12	2.09	0.5
C	2.38	2.19	2.14	2.05	0.5
D	2.33	2.26	2.11	2.11	0.5
**2'-dAdo/K^+/Zn^{2+}**					
A	2.32	2.09	2.42	2.13	0.11
D	2.29	2.13	2.39	2.19	0.19

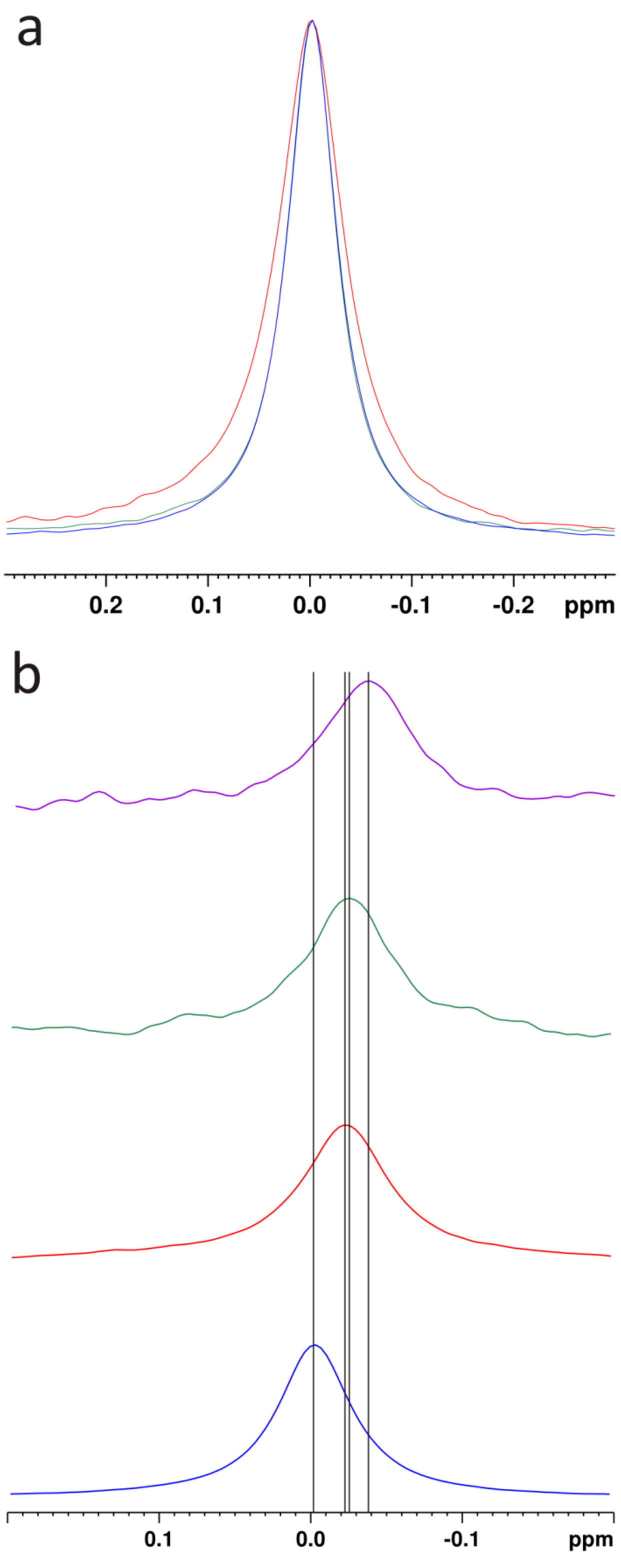

Figure S1. Comparison of ^{23}Na NMR spectra of solutions containing: (a) 5 mM NaCl (green), 5mM NaCl + 1 mM Ado (blue), and 5 mM NaCl + 0.3 mM PaSAHase (Enz, red); (b) 5 mM NaCl (blue), 0.3 mM Enz + 5 mM NaCl + 1 mM Ado ([Enz]/[Na^+]=1:16.7, red), 0.3 mM Enz + 0.6 mM NaCl + 1 mM Ado ([Enz]/[Na^+]=1:2, green), 0.3 mM Enz + 0.3 mM NaCl + 1 mM Ado ([Enz]/[Na^+]=1:1, purple). All samples were prepared in 50 mM Tris·HCl at pH 7.5.

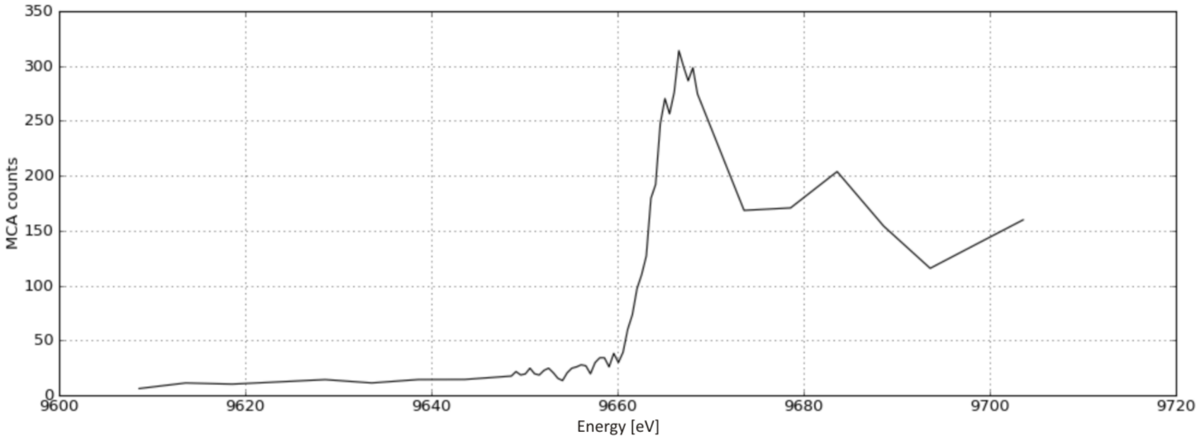

Figure S2. X-ray fluorescence spectrum recorded for PaSAHase sample co-purified with Zn^{2+} ions.
